# Exploring nurses’ perspectives on video consultations and the nurse–patient relationship: Findings from qualitative focus group interviews in a Danish cardiology outpatient setting

**DOI:** 10.1371/journal.pone.0339752

**Published:** 2026-08-11

**Authors:** Sissel Groth, Anne Brødsgaard, Signe Stelling Risom, Jens Dahlgaard Hove, Stine Rosenstrøm

**Affiliations:** 1 Department of Cardiology, Amager and Hvidovre University Hospital, Hvidovre, Denmark; 2 Department of Paediatrics and Adolescent Medicine, Copenhagen University Hospital Amager and Hvidovre, Hvidovre, Denmark; 3 Department of Obstetrics and Gynaecology, University Hospital, Amager Hvidovre, Hvidovre, Denmark; 4 Department of People and Technology, Roskilde University, Roskilde, Denmark; 5 Department of Public Clinical Health, Nursing and Healthcare, University of Aarhus, Aarhus, Denmark; 6 Department of Cardiology, Herlev and Gentofte University Hospital, Herlev, Denmark; 7 Institute of Nursing and Nutrition, University College Copenhagen, Copenhagen, Denmark; 8 Faculty of Health and Medical Sciences, University of Copenhagen, Copenhagen, Denmark; Cleveland Clinic, UNITED STATES OF AMERICA

## Abstract

**Background:**

Video consultations are increasingly implemented in healthcare, their integration into clinical practise remains challenging. Nurses’ uncertainty derived from internal and external influences may hinder the use of technology. Limited research has examined nurses’ experiences and perceptions related to the underuse of video consultations.

**Aim:**

This study aims to explore outpatient clinic nurses’ experiences with video consultations and to identify the organisational prerequisites they consider important for successful implementation of video consultations in outpatient clinics.

**Materials and methods:**

A hermeneutic qualitative exploratory approach was applied explore and interpret experiences and perceptions. Data were generated through two focus group interviews with 5 and 7 participating nurses in each group. The participants were recruited from two Danish cardiology outpatient clinics selected to ensure relevant clinical experience. Data were analysed using thematic analysis guided by Braun & Clarke.

**Results:**

Data provided insights into the perspectives of nurses, resulting in three main themes: *Video consultations must be used at the right time*, *Nursing care must remain as a relational practice*, and *Missing initiatives for enabling nurses to use video consultations*. Nurses experienced video consultations as both beneficial and challenging, shaped by tensions between efficiency demands, organisational conditions, and relational nursing values. Although video consultations offered flexibility and potential improvements in care delivery, nurses’ use depended largely on organizational support and integration into clinical workflows. Participants emphasised that high-quality nursing care is based on relational, embodied, and ethically responsible practices, which they perceived as potentially compromised in digital encounters. Organisational factors, including leadership, training, and clear implementation strategies, were identified as critical to promoting sustainable use.

**Public contributions:**

No Patient or Public Contribution.

## 1. Introduction

The rapid evolution of digital technologies has introduced new terminologies and reshaped healthcare delivery models [1]. Virtual health care has become a part of how care is delivered, received, and experienced [2]. In its guidelines and recommendations, the WHO highlights both client-to-provider and provider-to-provider telemedicine as key digital interventions for strengthening the healthcare system [3]. Increasing demand for outpatient services represents a global challenge, where digital solutions such as video consultations may help improve access and efficiency [4].

Although the COVID-19 pandemic accelerated the rapid adoption of virtual healthcare settings [2], sustaining these changes remains challenging [5]. Video consultations are increasingly embedded in healthcare digitisation and have the potential to improve care quality and patient accessibility [6,7].

However, implementation is not without difficulties. Nurses working with video consultations in cardiac care have reported feelings of insecurity derived from different internal and external influences, which may challenge the use of technology in health care [8]. Concerns about usability, especially for older patients, continue to represent a barrier for sustained implementation [7]. From the patient’s perspective video consultations are generally viewed positively. A review focusing on contextual factors found that patients often emphasise benefits such as timesaving and reduced need for travel [9].

Telehealth is characterised by a complex interplay of technological, social, and organisational factors, where successful implementation depends on the alignment between these elements [10]. As key actors within the healthcare system, nurses play a central role in shaping the use of video consultations. Their experiences therefore provide important insight into how this technology affects patient care, clinical work routines, and professional interaction [11]. Further, how nurses experience and adapt to this new form of communication to offer video consultations as politically intended in the health care system [12].

Ideally, virtual healthcare initiatives such as video consultations should be carefully planned, with barriers and facilitators identified prior to implementation or scaling [2]. This planning process supports delivering quality care and to maintain the relevance and sustainability of virtual care in an increasingly digital healthcare system [2].

Although video consultations are increasingly used in healthcare, few studies have explored nurses’ experiential and relational perspectives in cardiology outpatient contexts. Existing research provides limited insight into how nurses experience the use of video consultations and how it may influence nursing care and patient relationships. Further, which organisational prerequisites are needed for at successful implementation. Therefore, further research is needed to better understand nurses’ experiences with video consultations in cardiology outpatient clinics in order to identify and address prerequisites, support implementation in alignment with clinical priorities, and explore how video consultations may support or challenge relational aspects of nursing care for adult patients in outpatient settings.

## 2. The study

### 2.1 Aim and objective

This study aims to explore outpatient clinic nurses’ experiences with video consultations and to identify the organisational prerequisites they consider important for successful implementation of video consultations in outpatient clinics.

## 3. Materials and methods

### 3.1 Design and theoretical framework

Given the study’s objectives to shed light on outpatient clinic nurses’ experiences and perceptions, a qualitative exploratory research method was used [13]. The study adopts a hermeneutic approach to enable an interpretive understanding of outpatient clinic nurses’ lived experiences. Hermeneutics is appropriate because the aim is to interpret how meaning is constructed within these experiences [13]. The hermeneutic approach recognises that understanding is always situated and influenced by the researchers’ pre-existing knowledge and understanding, which is made explicit and reflected upon throughout the analytical process. Interpretation of the findings is developed through continuous movement between parts of the data and the whole, allowing meaning to evolve during analysis [13]. The reliability of this study was ensured through descriptions using Lincoln and Guba’s criteria for trustworthiness during the analysis process [14]. To ensure transparency and completeness in reporting, the study followed the Consolidated Criteria for Reporting Qualitative Research (COREQ) checklist. The checklist guided the study throughout its development, execution, and reporting phases [15].

### 3.2 Data collection

#### 3.2.1 Sampling and recruitment.

The study was carried out in a university hospital located in the capital region of Denmark, specifically within a cardiology outpatient clinic spread across two sites in Copenhagen. Two focus group interviews were conducted, one in each location familiar to the participants to create a comfortable contextually relevant setting and to avoid transport time for the participants. Different locations also allowed the study to capture potential variations in local practices and perspectives across the outpatient clinic sites. The focus group interviews were conducted to gather information from the different participants’ point of view [16]. Further, to understand and explain the meanings, beliefs, and cultures that influence the participants feelings, attitudes, and behaviours [16]. The groups were designed to include five to eight participants: small enough to allow each individual to participate fully and be heard, yet large enough to allow for varying opinions and perspectives [16].

The interview dates were chosen by the first and last author, and all nurses from the outpatient clinic working on the selected dates were invited to participate in the focus group interviews. In total 17 clinical nurses. The nurse managers ensured that information about the interviews was communicated, and that time was allocated within the outpatient nursing programs.

All outpatient clinic nurses were invited to participate in the focus groups. None were excluded. The participants were all female nurses working in cardiological clinical practice and knew each other from their daily work. Although the invited outpatient clinic nurses were relatively homogeneous, variations in participants’ digital experiences as well as attitudes and preferences were assumed variated. Exploring these differences was considered important to support a relevant subsequent implementation. Four invited nurses cancelled due to having scheduled time off, and one due to being too busy on the interview day (Table 1). All participants were knowledgeable about video consultations, having been introduced to the technology and trained on its use through a partner training session during the implementation process in 2021. This training included a brief technical introduction, instructions on scheduling video consultations, and operational guidance. A flowchart was created to help clinicians determine the suitability of video consultations. Although video consultations were prioritised for implementation at the time of the interviews, their use was optional in both outpatient clinics, and in-depth experience was not required.

**Table 1:** Participant characteristics.

Interview	Current position	Clinical experience from cardiac outpatient clinic practice	Cardiological field	Number of video consultations (estimated by the participants)
1 (Participant 1–7)	Registered nurses (n = 7) Advanced practice nurses (n = 0) Staff nurses (n = 0)	Mean 9 years Range1–15 years Median 10 years	Heart rehabilitation, heart valve patients, treatment for hypertension, arrhythmia, heart failure, endocarditis, anticoagulation treatment, and postpartum patients	From 1 to 50 (one participant had conducted approximately 200, as she had completed the virtual postpartum programmes in the outpatient clinic)
2 (Participant 8–12)	Registered nurses (n = 4) Advanced practice nurses (n = 0) Staff nurses (also RN) (n = 1)	Mean: 12 years Range: 4–22 years Median 8 years	Heart rehabilitation, heart valve patients, treatment for hypertension, arrhythmia, heart failure, and anticoagulation treatment	From 0 to 20

The participants’ experience with the use of video consultations was not a requirement for participation in the focus group interview, but instead the interest in sharing their thoughts on the use of video consultations.

All outpatient clinic nurses were equipped with headphones and a video camera connected to their computers in the consultation rooms. For video consultations, nurses connected with patients via an e-health platform, which required a link accessible through a smartphone, tablet, or computer. Other than connecting via a video link on the health platform, video consultations necessitated the same preparatory work as physical consultations. While video consultations were accessible to all participants, they had not been implemented to the same extent as physical or telephone consultations.

#### 3.2.2 Data sources/collection.

The first author was a registered nurse with a Master of Science in clinical nursing and the last author was a registered nurse with a Ph.D. First and last author who were responsible for conducting the interviews, participated in a pre-understanding interview [17] conducted by the third author. This preunderstanding interview aimed to reveal the author’s preunderstanding of the topic, thereby promoting explicitness and transparency during the data collection and processing stages [17]. Both authors possess an in-depth experience as clinical registered nurses in the cardiological field. The first author had no clinical experience with outpatient clinic treatment or virtual consultations. The last author has long-standing experience with outpatient clinic treatment and virtual consultations. The first author moderated both interviews and thus had the responsibility to facilitate discussions and exchange of ideas between the participants [16]. The last author ensured that all themes were covered through the questions from the interview guide (Table 2) and that all participants could express their views [18]. Physical interviews took place in undisturbed meeting rooms located within the participants’ clinical settings to ensure privacy and minimise interruptions. The interviews were conducted in the afternoon, at a time when most participants had no scheduled patient consultations. Conducting the interviews physically enabled attention to body language and interaction patterns while supporting open discussions and nuanced exchanges within the relatively homogeneous group. The interviews were conducted in the participants’ native language (Danish).

**Table 2:** Interview guide.

Themes	Questions
Attitude towards IT solutions	What are your perceptions of physical consultations being replaced by virtual ones? Can you unfold pros and cons when using digital solutions such as video consultations in the outpatient clinic?
Functional and clinical needs	When is a consultation successful to you? What does a successful consultation mean to you?
Emotional needs	How do you feel during a consultation? What do you value? How do you feel about physical consultations being replaced with virtual ones?
Social needs	How would you describe your relationship with patients during a consultation?

The interview guide was created based on a systematic literature review, clinical experiences, and qualifications of the first and last authors. Although the interview guide was not formally pilot tested, it was thoroughly discussed and refined among the first and last author prior the interview. The interview guide served as an inspiration to unpack the participants’ experiences and perceptions. It was created to uncover what was deemed valuable in patient-provider relationships, explore different perspectives and requirements of physical consultations, identify perceived limitations, and reflect upon the potential of video consultations. It was emphasised that reaching a consensus was not the goal; rather, the aim was to encourage the participants to share their experiences, perspectives, and curiosities with one another [18]. All themes and related questions were thoroughly discussed. While the questions did not always follow the interview guide’s chronological order, the guide was instrumental in ensuring that all themes were illuminated to sufficient depth and nuance. To support a safe and confidential discussion environment, the interviews were initiated with a short informal chat followed by

a clear and transparent framing of the study aim before diving into the topic. The intended use of the data and the procedures related to anonymity and confidentiality were clarified. Verbal and nonverbal facilitation techniques were used throughout the interviews to acknowledge and accommodate difficult or conflicting emotions and viewpoints. The moderators applied affirmative communication and active listening strategies and deliberately avoided interrupting participants. Further progressively focusing the participants’ attention on the topics of most significant interest [18]. The facilitator of the interviews tried to take advantage of the group homogeneity in terms of educational background and current context in facilitating open communication, exchange of ideas, and a sense of safety in expressing conflicts of concerns [16].

Each session lasted approximately 90 minutes and was audio-recorded. The interviews were transcribed verbatim by the first author. The names of the participants were anonymised and replaced with numbers for analysis and reporting. For dissemination purposes, the transcribed data used to support the result were translated by the first author. Translation accuracy was verified by the first and last author. Data collection was considered sufficient and data saturation achieved, when the interviews provided rich and nuanced insight relevant to the research aim and to the context of video consultation implementation.

### 3.3 Ethical considerations

Participation in the study was voluntary and took place during working hours. Both written and oral information about the study was provided to the participants, and written informed consent was obtained from all participants before starting the interviews. The study did not require approval from Danish research ethics committees (Journal No. F-23075094), since the study does not involve experiments on live-born human individuals, human gametes, experiments with drugs, testing of medical devices or similar procedures.

### 3.4 Analysis

The transcribed interviews were analysed using thematic analysis, for systematically identifying, organising, and gaining insights into patterns of meaning or themes across the interview data [19]. The thematic analysis acknowledges the active role of the researcher in identifying patterns/themes, deciding on their relevance, and reporting them to the reader [19]. The process begins as researchers identify potential patterns of meaning and issues of interest that can occur during data collection. The analysis involves constantly moving back and forth between the entire dataset, the coded extracts of data being analysed, and the ongoing analysis of the data being produced [19]. The hermeneutic approach was used to facilitate a deeper understanding of the interview text by alternating between parts of the data and the dataset as a whole, interpreting the findings by continually questioning their meaning [13]. The analysis followed the six phases of thematic analysis recommended by Braun and Clarke (2006), as we wanted to work systematically with data while interpreting it in terms of meaning, context and understanding. Braun og Clarke offer a systematic structure for analysing qualitative data:

Phase 1: *Familiarising oneself with the data* implied transcribing the data as an interpretative act, reading and re-reading the data, and searching for meanings and patterns.
Phase 2: *Generating initial codes* entailed a systematic examination of the entire dataset, paying full and equal attention to each item and identifying aspects that might form the basis of repeated patterns. The coded data were organised into meaningful groups relative to the study’s aim.
Phase 3: *Searching for themes* involved sorting the initial codes into potential themes, with all relevant coded data extracts collated within these identified themes. A mind map was used to create an overview and understand the connections between themes, using separate pieces of paper with the name of each code and brief descriptions to experiment and organise themes. Codes that did not seem to belong anywhere were collected in a ‘miscellaneous group’.

First author completed phase 1–3.

Phase 4: *Reviewing themes* required refining the themes through critical reviewing. First, we considered whether the codes formed a coherent pattern and adequately captured the perspectives within the coded data. Second, reading alternating sections of the text generated new understandings of the individual sections and the text as a whole. The refinement process continued until no substantial new insights were added.
Phase 5: *Defining and naming themes* involved identifying the ‘essence’ of each theme and determining what aspect of the data each theme captured.

Researcher triangulation was conducted by the first and last author to validate the analysis in phase 4–5. Coded data were used as a basis for reflective discussions between the first and last author about content and meaning, and consensus was reached through these discussions to achieve a nuanced understanding of the data. The themes were not understood as emerging directly from the data but were actively generated through the researchers’ interpretative engagement with patterns of shared meaning across the coded dataset.

Phase 6*: Producing the report* entailed reporting the reasons for theoretical, methodological, and analytical choices, describing the coding and analysis processes in sufficient detail to capture the essence of the points without unnecessary complexity, and providing a thorough description of the context to ensure transferability. Quotations from the interview text were used to illustrate the analytical process, supporting the transparency and trustworthiness of the findings and the authors’ interpretations of the data (Table S3 in the appendix in S1 File). In this phase, the entire research team completed peer debriefing and researcher triangulation to validate the interpretation.

## 4. Results

Three themes were developed through the interpretative process based on 12 codes: 1) *Video consultations must be used at the right time*, was developed from codes describing how nurses assessed the appropriateness and potential value of video consultations in specific clinical situations, including relative involvement, cross-sectoral collaboration, opportunities for new observations, and nurses’ professional judgement in selecting consultation types 2) *Nursing care must remain as a relational practice* was developed from codes reflecting concerns about how video consultations influenced relational and professional aspects of nursing practice. These included concerns about the patient relationship, reduced work satisfaction, the complexity of nursing practice, and the perceived risk that video consultations could devalue nursing competencies. 3) *Missing initiatives for enabling nurses to use video consultations* was developed from codes related to organisational, technical, and practical barriers to implementation. These included the perceived flexibility of telephone consultations compared with video consultations, lack of organisational and technical support, and the need for knowledge and competencies in facilitating video consultations. These themes are presented in Table S3 in S1 File, supported by illustrative, representative, and rich quotations.

### 4.1 Video consultations must be offered at the right time

A common view was an overall concern about the increasing use of technological solutions in outpatient clinic treatment and care activities, which illustrates an underlying tension between technological expansion and professional discretion in clinical decision-making. In general, the participants saw both possibilities and limitations in the different types of consultations: physical, telephone, and video. When choosing among these types, both patients’ and nurses’ preferences were considered important factors. One participant noted:

*‘I think that those patients I speak with over the telephone are the same patients I would offer a video consultation’. (Participant 12)*

The participants acknowledged the nurses’ role in initiating the use of technology and how the different consultation types are presented to patients. Positioning nurses as gatekeepers highlights their agency in mediating technological implementation. All agreed that video consultations should be carried out if patients demand or prefer them:

*‘Whether it is the choice between attendance [physical consultation] or video consultation, I think they should be allowed to choose (…) I think it would be a shame if they were forced to do video just because we believe it is smart’. (P9)*

The participants expressed that the first meeting with a patient should preferably be a physical consultation because it establishes the relationship. This reflects an implicit hierarchy of consultation forms, where physical presence is constructed as foundational for relationship-building. They acknowledged that subsequent nursing consultations could be conducted effectively via video, offering value to some patients. The participants also noted that video consultations could be an efficient means of collecting additional information about the patient’s daily life, thus serving as a good alternative to telephone consultations by supporting their memory of the patient:

*‘I see their faces on the screen, and I don’t always remember what they look like if I speak with them on the phone. (…) You can get some information about their daily lives in a way. (…) Yesterday, one was smoking while we were having the consultation; she only took one drag of the cigarette, but at least it was enough for me to remember asking her to consider stopping smoking’. (2)*

The participants highlighted that the choice of consultation type (physical, telephone, or video) should be tailored to the specific needs of each patient:

*‘I had an old lady who couldn’t get out of her bed. Her daughter then helped her join a video consultation, and then I spoke with both. It would be troublesome for her to get to the hospital for a physical consultation, but on the other hand, she is dependent on her daughter to help her. She could never manage it by herself’. (P2)*

However, others emphasised that the use of video consultations could initiate a stronger cross-sectoral collaboration by including home care nurses and general practitioners in these consultations to collectively support vulnerable patients. Video consultations could also be used to involve relatives who might not attend physical consultations. There was a consensus that involving the primary care sector and relatives could be highly beneficial for patients but would require further development and integration into care and treatment workflows to monitor specific care and treatment tasks and system access. The findings indicate that increased use of video consultations depends on aligning technological options with nurses’ clinical judgement, patient preferences, and organisational structures that support flexible and coordinated care delivery.

### 4.2 Nursing care must remain as a relational practice

The participants expressed ambivalence regarding the justification for video consultations compared to physical consultations, framing them primarily through an efficiency-driven logic rather than clinical or relational rationale:

*‘It’s an efficiency improvement system [the video consultation], that’s how I see it. It’s because we must achieve several things in the shortest possible time. We do this because we can (…) it’s not necessarily a good thing’. (P1)*

The quote illustrates how video consultations were received as embedded in a broader organisational rationality of optimisation and time-efficiency, which was not necessarily aligned with nurses’ professional judgement of what constituted “good care”. The perceived nurse shortage across the healthcare system influenced the nurses’ perceptions and perspectives on implementing video consultations. Nurses are aware that resources must be distributed and utilised wisely, and they were concerned that patients should benefit from all the time allocated. The participants felt that the political agenda and arguments supporting telemedicine, and the benefits of video consultations had not been clearly communicated by the department or hospital management, leading to reduced motivation to adopt video consultations. Furthermore, the participants agreed that video consultations do not save time but rather degrade the quality of nursing care by challenging traditional patient interactions. The participants feared that video consultations would diminish their job satisfaction by reducing the relational dimensions of their clinical work:

*‘If the management said all consultations must be virtual, then I know I would find it boring’. (P8)*

As the quote illustrates, the participants not only fear reduced variation in work tasks, but also how video consultations were associated with a potential loss of professional meaning derived from direct patient interaction and relational engagement. Nonetheless, they were aware of the necessity of keeping pace with technological advancements in society to meet patients’ evolving demands and expectations. The participants also expressed the need to acquire new knowledge and skills to adapt effectively.

The participants were strongly committed to providing nursing care that ensures and maintains patient empowerment, safety, and security. More specifically, all participants felt a great responsibility to support and encourage their patients to manage their heart disease effectively. One participant expressed:

*‘We must educate the patients to react to symptoms, take care of themselves, and prevent (…) the aggravation of heart failure. We must guide them in weight loss, diet, physical activities, and lifestyle risk factors, explaining the importance of taking the prescribed medicine’. (P4)*

The nurse’s professional identity seems deeply grounded in embodied, relational and ethically orientated care. A common view was that the desired achievements in nursing consultations required physical contact to establish a meaningful relationship with the patients. This indicates that resistance to using video consultations is not merely practical but rooted in normative understandings of what constitutes ‘good nursing’. From the participants’ perspective, a meaningful relationship required ‘good chemistry’, empathy, and intuition:

‘*The patients’ understanding does not only require explanation, but it also demands that you really get under their skin, and then we try to ensure the patients’ feeling of safety. (…) We enlighten their hearts (…) we really have to listen to the patients and embrace them’. (P5)*

A participant described feeling that video consultations diminished the value of her education and clinical experience by undercutting her professional competencies:

*‘I feel it’s a devaluation of my education and my life experience as a nurse (…) intuition and empathy’. (Participant 1)*

This indicates that relational care may be constructed as embodied and intuitive, which may be difficult to translate into digital formats.

The participants felt a strong sense of responsibility towards their patients and thus had to advocate for the most suitable consultation type. The findings indicate that ambivalence towards video consultations reflects a tension between efficiency-driven organisational logics and nurses’ relational and embodied understanding of “good care”, and highlights nurse-identified organisational prerequisites for increased use, aligning digital solutions with professional identity clear communication and organisational support.

### 4.3 Missing initiatives for enabling nurses to use video consultations

The participants emphasised that management had an important role in directing how nurses and other healthcare professionals should prioritise video consultations. This underscores that implementation is not only an individual responsibility but organisationally embedded. Some participants were concerned that video consultations would only increase the nurses’ workload with additional tasks, such as teaching patients to use the technology, dealing with technical issues, and meeting a demand for greater availability and flexibility. When patients needed earlier appointments than scheduled, participants found it more straightforward to offer a telephone consultation rather than a video consultation because it felt easier to integrate into an already busy schedule as they were perceived as more adaptable to ad hoc clinical demands:

*‘I had a patient in a video consultation yesterday whom I wanted to see again soon. However, I had no appointments available for either physical or video consultations. Instead, I had to offer a telephone consultation, where I could call her during the day without a specific time’. (P2)*

The organisation of the outpatient clinics and scheduling of patients sometimes acted as barriers to video consultations because only telephone consultations could be flexibly added as an extra option. This points to a misalignment between technological possibilities and infrastructural realities. The participants stated that management wished for video consultations to be offered equally alongside physical and telephone consultations in the outpatient clinic. Furthermore, management needed to prioritise time for education and the development of digital competencies to ensure high-quality video consultations. However, this managerial ambition was experienced in tension with limited clinical time and competing demands, indicating a gap between policy expectations and the situated realities of implementation:

*‘It is difficult to make changes when there isn’t time for it. (…) What is it, really? What should I do when I have a video [consultation]? It’s very rare that we have enough time, and then you don’t take ownership of it because it [the implementation process] feels a little sloppy, and then you become sloppy, too’. (Participant 12)*

At the beginning of the video consultation implementation process, the participants received partner training to become familiar with the technology. However, they mentioned that it was too brief:

*‘It [the implementation of video consultations] worked very well, but it should have lasted a little longer. To implement something takes not only a day. It takes months to maintain changes. (P12)*

After the partner training, the participants felt they lacked support in addressing questions and challenges related to video consultations. This lack of sustained scaffolding meant that responsibility for implementation was largely transferred to individual nurses, which in turn shaped the motivation and ownership in practice. They argued that adequate resources must be allocated to training staff to maintain motivation, given that outpatient clinic nurses’ time was scarce and utilised to the maximum, which indicates that motivation is structurally conditioned rather than individually driven.:

*‘There was a long period last summer where it didn’t work at all (…) maybe it’s different now, but then I stopped planning video consultations because I ended up calling on the telephone anyway. (…) If you have to discover yourself, that it doesn’t work [the video link] when you are sitting with the patient, then the motivation disappears’. (P11)*

The participants explained that they often used telephone consultations when video links did not work, leading many to prefer telephone consultations. It was a commonly held belief that video consultations require different communicative competencies to maintain good relationships with patients compared to telephone or physical consultations. The participants explained how video consultations demand alternative approaches to understanding patients’ perspectives and symptoms when not all senses can be engaged in the overall assessment of the patient. Additionally, facilitating video consultations requires different approaches compared to physical consultations:

*‘... it’s a bit of an art to give time and breaks when you communicate through a video consultation in contrast to how we normally communicate in the physical consultation’. (P2)*

Beyond the need for education on conducting video consultations for nurses, the participants also highlighted the necessity of educating patients on how to use video consultations effectively. In current practice, patients’ technical support needs fall on the nurses, with time allocated for this support detracting from the consultation time, thus necessitating a prioritisation of technical support over health-related discussions. This illustrates how telemedicine implementation redistributes not only tasks, but also the boundaries of clinical responsibility in practice. Looking ahead, the participants anticipated that telemedicine would face far fewer challenges and barriers. However, this expectation of “automatic” adoption coexisted with continued uncertainty regarding technological reliability and clinical readiness:

*‘I think it will change automatically. Seniors, including myself, have a little fear of it [video consultations] because we didn’t grow up with it. (…) I’m afraid that the technology won’t work’. (P7)*

Despite anticipating that the demand from future patients may lead to the natural adoption of video consultations, the participants agreed on the importance of being proactive in preparing themselves for these future demands. The findings suggest that increased use of video consultations depends, from the nurses’ perspectives, on aligning managerial expectations with clinical realities particularly by addressing time constraints, limited implementation support, and the fit between digital workflows and everyday nursing practise.

## 5. Discussion

The findings provided a nuanced understanding of the factors that can affect the use of video consultations, suggesting that their limited integration is not only a question of technology, but equally a matter of contextual fit, professional judgment, and organisational support. When these factors are not adequately addressed, it seems they contribute to the continued limited use of video consultations in outpatient clinic settings.

The finding that ‘*Video consultations must be used at the right time*’ highlights that nurses do not merely perceive barriers and benefits but actively decide whether video consultations are clinically meaningful, reflecting a situated, context-dependent decision-making process. The finding also underscored nurses’ recognition of the challenges and limitations, as well as the potential benefits of video consultations for both nurses and patients. In this study, nurses appreciated the convenience of video consultations offered to patients and their families by saving transportation time and allowing more flexible scheduling. This aligns with previous findings identifying video consultations as time – and cost saving [20]. However, the present study suggests, that convenience is insufficient to ensure adoption, as it must be balanced with clinical workflow and relational considerations. Together, these results point to a need to rethink the decision-making process, potentially moving towards a more patient-centred approach and shared decision-making rather guided by professional assessment. The participants also viewed video consultations as an opportunity to enhance cross-sectoral collaboration with home care nurses and general practitioners, supporting the exchange of knowledge for improved patient care. Similarly, other studies have highlighted the benefits of cross-sectoral video consultations in contributing to patient-centred care, the clarity of roles, treatment continuity, and patient satisfaction [21,22].

Some nurses noted that video consultations offered them opportunities to gather additional information about their patients by seeing them in their home environments virtually, in contrast to telephone consultations. This way nurses could observe patients’ surroundings on screen and use these observations in their clinical assessment. Additionally, video consultations enable the identification of non-verbal cues, which is not feasible with telephone use alone [20]. The nurses acknowledged their role in initiating video consultations, and actively selecting which patients to offer them to, based on what they deemed best for their patients, potentially positioning patients in a more passive role (Molina-Mula & Gallo-Estrada, 2020).Furthermore, the nurses acknowledged that patients’ needs, and demands are highly important when choosing the type of consultation. The nurses’ clinical experience working in a cardiology outpatient clinic setting varied, which appeared to influence their use of video consultations, suggesting that experiential and contextual factors shape adaption. In contrast, one study found no correlation between age or seniority and the intention to use telenursing and video consultations [23].

The insight that *Nursing care must remain as a relational practice* revealed the importance that the nurses placed on their relationship with patients to deliver high-quality care. The nature of the clinician-patient relationship directly affects the quality of care [24]. It has been noted that a good relationship initiated by the expert professional directs knowledge, professional experience, and clinical skills toward the specific needs of each patient [24]. These findings align with the nurses’ perceptions in this study, emphasising the continued importance of physical interaction.

Professional judgment, seen as an interplay of senses, involves being present with one’s entire personality and professionalism [25]. The nurses found achieving this presence challenging without physical interaction. In response, they often prioritise physical consultations for first meetings and consciously select consultation formats that they perceive as better suited to relational assessment, reflecting a strategic prioritisation of relationship-building as a prerequisite for subsequent digital interaction. Referencing Løgstrup [26], care ethics suggest that humans are inherently connected and have a moral obligation to act in others’ best interests and care for the lives entrusted to them [27]. The nurses in this study expressed concerns about fulfilling their ethical and moral responsibilities through video consultations. This has important theoretical implications, as relational nursing theory does not fully account for digital forms of care, including their limitations and conditions, thereby challenging assumptions underlying digital implementation in healthcare.

It has been shown that practising care by telephone is complex due to the inability to offer physical reassurance, such as placing a comforting hand on the patient [28,29]. The nurses in this study expressed that video consultations challenge their clinical intuition and empathy, potentially leading to a devaluation of their professional experience. This concern has also been identified as significant in adapting to new technologies [10].

The finding of ‘*Missing initiatives for enabling nurses to use video consultations’* revealed that organisational leadership, prioritisation and strategic direction are crucial in successful digital implementation. Nursing leadership plays a central role in translating digital health technologies into sustainable clinical practice by ensuring alignment between organisational goals, resources and workflows. Managerial encouragement, structured training, and the provision of resources were found to be imperative for enabling nurses to employ video consultations [23].

One study identified the willingness of institutional forces at both societal and organisational levels to invest in strategic purpose and funding as the driving force behind successful implementation [9]. Although participants in this study were introduced to video consultations through the same implementation process, their levels of use varied. Rogers [30] noted that individuals within a social system adopt innovations at different rates, highlighting the need for tailored implementation and follow-up strategies. The participants emphasised the need to develop technological competencies before offering video consultations. The focus on enhancing competencies and relatedness is supported by several studies [28,31]. Reflecting on theory and their practice allows nurses to redefine their professional roles to provide remote patient care while ensuring the delivery of high-quality care [28]. Moreover, enhancing competencies is essential for fostering a feeling of autonomy, which is vital for intrinsic motivation in humans [31]. Successful implementation relies on motivated individuals to come up with innovative solutions to solve problems as they arise [32]. Therefore, sustainable implementation of video consultations requires more than initial training. It demands iterative organisational learning processes, involving continuous competency development, structured reflection on practice and active involvement in redesigning workflows. Stakeholders to ensure engagement, process mapping to delineate the inputs, outputs, and steps of a specific change initiative, and problem-solving [32].

Overall, this study indicates that successful integration of video consultations requires a combined implementation strategi which implies organisational commitment and resource allocation, adaptive leadership and communication and continuous co-creation with clinical staff to ensure that digital solutions remain aligned with both clinical practice and nursing values.

### 5.1 Strength and limitations of the work

#### 5.1.1 Strength.

This study has several strengths. A significant strength was the relatively large number of participations, representing over half of the outpatient clinic nursing staff (in total 17). This substantial representation enhances the relevance of the findings and provides rich insight into nurses’ perspectives on video consultations within the specific clinical context. Additionally, the inclusion of nurses from the department in which video consultation were to be implemented offered valuable practice-based insight that may inform future educational and organisational initiatives and strategies.

Methodological rigour further strengthens the study. Uncovering the interviewers’ preconception through preunderstanding interview sharpened the awareness of tacit knowledge. At the same time, familiarity with the department, the culture, and the organisation enabled the interviewer to probe further and unravel the nuances in statements. To ensure credibility, the choice of facilitator was based on having the least familiarity with the outpatient clinic and its nursing staff. The interview process and techniques were consistent across the interviews, focusing on exploring different subjects and perspectives until data saturation was achieved. To ensure the study’s transferability, detailed descriptions of the data were provided. Rich descriptions of the qualitative methodological approach strengthened the study’s dependability, enabling the exploration of the research aim and showing that the findings were consistent. Coding accuracy and reliability were measured by the research team. Further, the whole research team discussed coding for sufficient data saturation. A high level of reflexivity throughout the analytical process through continuous discussions among the authors strengthened the study’s confirmability.

#### 5.1.2 Limitations.

This study also has several limitations. A key limitation is that the interviewers was part of the same department as the participants, which may have challenged the objectivity in conducting the interviews. Possible response bias may exist due to the shared workplace and pre-existing relationships between the interviewers and participants. However, efforts were made to reduce this risk by thoroughly informing participants at the beginning of the interviews about anonymity and confidentiality, and by emphasizing that the interviews would contribute important knowledge to the planning and organising of the further implementation process.

Finally, the study’s transferability may be limited by the relatively small sample size in a Danish context and the inclusion of participants from only one department, albeit across two sites. Although the findings provide valuable contextual insights, they may not be fully generalisable to other settings.

### 5.2 Recommendations for further research

The findings of this study indicate unmet needs and suggest areas that may lead to better implementation of video consultations in outpatient clinic settings. Based on this knowledge, recommendations for guidelines can be proposed. To ensure successful and sustainable implementation, management must foster a motivated workforce capable of developing innovative solutions to emerging challenges. Management must ensure that staff members possess the knowledge, skills, and attitudes to support the integration of video consultations on par with telephone and physical consultations. Thorough and systematic training of nurses is necessary, along with a sustained focus on identifying and addressing the opportunities and challenges presented by the technology in relation to patient care. Future research should focus on practice-oriented approaches to implementation. Comparative studies examining differences in patient outcomes, communication quality, and workflow efficiency between video, telephone, and physical consultations would provide valuable insights into the most appropriate use of each consultation format. In addition, interventional studies evaluating the effect of structured nurse training programs, implementation strategies, or technological support initiatives could help identify effective methods for strengthening staff competencies and confidence in video consultations. Multi-center studies across outpatient clinics and healthcare settings are also recommended to explore organisational differences.

## 6. Conclusion

In conclusion, the nurses in this study experience video consultations as both beneficial and challenging, shaped by a tension between efficiency demands, organisational conditions, and relational nursing values. In practise, successful use of video consultations requires flexible integration into clinical workflows, alongside targeted support for nurses in managing both communicative and technical aspects of virtual care. The outpatient nurses emphasise that high-quality care is grounded in relational, embodied, and ethically responsible practice, which they fear may be weakened through digital consultation forms.

From a policy and organisational perspective, the findings highlight the need for sustained investment in training, clear implementation strategies, and leadership that actively aligns digital initiatives with clinical realities and professional nursing values.

Overall, sustainable digital transformation in outpatient care depends on ensuring that technological development supports rather than undermines the relational and ethical foundations of nursing practice, which requires active support, reflection, and prioritization from both leaders and educators.

## Supporting information

S1 File Table S3 Example of coding. (DOCX)
