# Supplementary material for: Exploring nurses’ perspectives on video consultations and the nurse–patient relationship: Findings from qualitative focus group interviews in a Danish cardiology outpatient setting
Source: PLoS One. 2026 Aug 11;21(8):e0339752. doi: 10.1371/journal.pone.0339752 (PMC13460611; doi:10.1371/journal.pone.0339752)
Supplement: Supplementary file 1 [file pone.0339752.s001.docx]

# **Supporting Information**

## Appendix

## Table S3 Example of coding

| **Quote** | **Codes** | **Themes** |
| --- | --- | --- |
| *‘I really want others to join too (…) home counsellors, primary nurses, or relatives for that matter’.* | Relative involvement  Cross-sectoral collaboration  Video consultations contribute to new observations.  The consultation type is picked out by the nurse. | Video consultations must be used at the right time. |
| *‘I had an old lady who couldn’t get out of her bed. Her daughter then helped her join a video consultation, and then I spoke with both. It would be troublesome for her to get to the hospital for a physical consultation, but on the other hand, she is dependent on her daughter to help her. She could never manage it by herself.’* |  |  |
| *‘I see their faces on the screen, and I don’t always remember what they look like if I speak with them on the phone. (…) You can get some information about their daily lives in a way. (…) Yesterday, one was smoking while we were speaking, while we were having the consultation; she only took one drag of the cigarette, but at least it was enough for me to remember asking for her to consider stopping smoking’.* |  |  |
| *‘I think that those patients I speak with over the telephone are the same patients I would offer a video consultation’.* |  |  |
| *‘It’s an efficiency improvement system [the video consultation], that’s how I see it. It’s because we must achieve several things in the shortest possible time. We do this because we can (…) it’s not necessarily a good thing’.* | Technology is not necessarily a good thing for the relationship with the patient.  Reduced work satisfaction.  Nursing is a complex practice.  Video consultations may devaluate nursing competencies. | Nursing care must remain as a relational practice. |
| *‘If the management said all consultations must be virtual, then I know I would find it boring’.* |  |  |
| *‘The patients’ understanding does not only require explanation, but it also demands that you really get under their skin, and then we try to ensure the patients’ feeling of safety. (…) We enlighten their hearts (…) we really have to listen to the patients and embrace them’.* |  |  |
| *‘I feel it’s a devaluation of my education and my life experience as a nurse (…) intuition and empathy’.* |  |  |
| *‘I had a patient in a video consultation yesterday whom I wanted to see again soon. I had no appointments available for either physical or video consultations. Instead, I had to offer a telephone consultation, where I could call her during the day without a specific time’.* | Telephone consultations can be used more flexibly than video consultations.  Lack of organisational support  Lack of technical support  Knowledge of facilitating video consultations | Missing initiatives for enabling nurses to use video consultations. |
| *‘It [the implementation of video consultations] worked very well, but it should have lasted a little longer. To implement something takes not only a day. It takes months to maintain changes’.* |  |  |
| *‘There was a long period last summer where it didn’t work at all [the technique] (…) maybe it’s different now, but then I stopped planning video consultations because I ended up calling on the telephone anyway’.* |  |  |
| *‘... it’s a bit of an art to give time and breaks when you communicate through a video consultation in contrast to how we normally communicate in the physical consultation’.* |  |  |
