# Supplementary material for: Exploring nurses’ perspectives on video consultations and the nurse–patient relationship: Findings from qualitative focus group interviews in a Danish cardiology outpatient setting
Source: PLoS One. 2026 Aug 11;21(8):e0339752. doi: 10.1371/journal.pone.0339752 (PMC13460611; doi:10.1371/journal.pone.0339752)
Supplement: Supplementary file 2 [file pone.0339752.s003.docx]

**Response to reviewers**

| Journal Requirements:  Please ensure that your manuscript meets PLOS ONE's style requirements, including those for file naming | We have revised the manuscript to meet style requirements. |
| --- | --- |
| Journal Requirements:  Please state what role the funders took in the study | We have stated that *the funders had no role in study design, data collection and analysis, decision to publish, or preparation of the manuscript* |
| Journal Requirements:  When you resubmit, please ensure that you provide the correct grant numbers for the awards you received for your study in the ‘Funding Information’ section. | Thank you for this comment. There is no grand number for these funds. |
| Journal Requirements:  We note that your Data Availability Statement is currently as follows: All relevant data are within the manuscript and in Supporting Information files. | Thank you for this comment, we will ensure that. |
| Journal Requirements:  We note that the main manuscript consists of interview transcripts. Can you please confirm that all participants gave consent for interview transcript to be published? | I confirm that all participants gave consent for interview transcript to be published. Please see the ethic section. |
| Additional Editor Comments:  Insufficient clarity in the research gap, aim, and overall focus | Thank you for this comment. The introduction has been thoroughly updated, including the research gap, aim, and overall focus |
| Additional Editor Comments:  Limited methodological transparency (sampling, data collection, analysis) and incomplete alignment with COREQ standards | Thank you for this comment, which has been elaborated. See the responses below to reviewer 1's comments.  COREQ checklist has been reviewed and updated. |
| Additional Editor Comments:  Predominantly descriptive results with limited interpretive depth | Results have been elaborated with more interpretive depth. See the responses below to reviewer 1's comments |
| Additional Editor Comments:  Repetition and lack of precision in parts of the abstract, introduction, and discussion | Thank you for this comment. We have reformulated the sections to be more clarified.  Se the answers below. |
| Reviewer #1 – Title and abstract  The “Materials and methods” section in the abstract could more succinctly indicate the theoretical approach and sampling rationale. | Thank you for this suggestion. We have revised the “Materials and methods” section in the abstract to more succinctly clarify the theoretical approach underpinning the study and the rationale for the sampling strategy:  *A hermeneutic qualitative exploratory approach was applied to explore and interpret*  *experiences and perceptions. Data were generated through two focus group interviews with 5 and 7 participating nurses in each group. The participants were recruited from two Danish cardiology outpatient clinics selected to ensure relevant clinical experience. Data were analysed using thematic analysis guided by Braun & Clarke.* |
| Reviewer #1 - Title and abstract  Include precise information on the analysis method (e.g., “thematic analysis guided by Braun & Clarke”). | We have revised the abstract to include a more precise description of the analysis method by explicitly stating that the *data were analysed using thematic analysis guided by Braun & Clarke.* |
| Reviewer #1 - Title and abstract  Avoid repetition of aims; integrate the second sentence into a single cohesive aim statement. | Thank you for this constructive comment. We have revised the aim: This study aims to explore outpatient clinic nurses’ experiences with video consultations and to identify the organisational prerequisites they consider important to increase the usage of video consultations in outpatient clinics. |
| Reviewer #1 - Title and abstract  Mention the number of focus groups and participants directly in the abstract for transparency. | Thank you for this helpful suggestion. We have revised the abstract to explicitly state the number of focus groups and participants to improve transparency and clarity:  *Data were generated through two focus group interviews with 5 and 7 participating nurses in each group.* |
| Reviewer #1 – Keywords  The selected keywords are appropriate; consider adding “telehealth” or “telemedicine” to align with global terminology and indexing conventions. | Thank you for pointing the global terminology. We have added both “telehealth” or “telemedicine”. |
| Reviewer #1 - Introduction  The introduction is rich but somewhat dense. Consider tightening for flow and removing minor redundancies (e.g., overlapping statements about the pandemic’s influence). | Thank you for this helpful comment. We have reformulated the introduction in general to improve overall flow and readability by tightening the text and removing repetitions, including overlapping statements.  ‘Virtual healthcare’, ‘video consultations’ and ‘digital technologies’ were repeated many times, which has been attempted to be formulated more comprehensively and clearly. We have formulated it so that there is progression in flow: technology, challenge, implementation, perspectives and needs. |
| Reviewer #1 - Introduction  The final paragraph should more clearly articulate the gap in existing knowledge leading to this study (e.g., “Few studies have explored nurses’ experiential and relational perspectives in cardiology outpatient contexts…”). | Thank you for this comment. We have revised the final paragraph to more clearly articulate the existing knowledge gap, particularly the limited research exploring nurses’ experiential and relational perspectives on video consultations in cardiology outpatient settings:  *Although video consultations are increasingly used in healthcare, few studies have explored nurses’ experiential and relational perspectives in cardiology outpatient contexts. Existing research provides limited insight into how nurses experience the use of video consultations in clinical practise, including both the practical and organisational barriers to implementation and the ways video consultations may influence nursing care and patient relationships. Therefore, further research is needed to better understand nurses’ experiences with video consultations in cardiology outpatient clinics in order to identify and address barriers, support implementation in alignment with clinical priorities, and explore how video consultations may support or challenge relational aspects of nursing care for adult patients in outpatient clinic settings.* |
| Reviewer #1 - Introduction  Clarify the conceptual grounding: Is the study primarily exploring perceptions and relational care or implementation barriers? Both are present but could be better integrated. | Thank you for this constructive comment.  We have revised and clarified the link between video consultations and their importance of patient relationships and nursing care. Further the lack of research, which is essential to accommodate and handle the experienced barriers in implementation:  *Existing research provides limited insight into how nurses experience the use of video consultations in clinical practice, including both the practical and organisational barriers to implementation and the ways video consultations may influence nursing care and patient relationships.* |
| Reviewer #1 – Aim and objectives  The aim is clearly stated but repeated in several sections (abstract, methods, and introduction). Condense to one consistent phrasing. | Thank you for this comment. The aim has been reformulated and ensured that it is consistent throughout the manuscript |
| Reviewer #1 – Aim and objectives  Consider adding a secondary objective explicitly addressing “identification of organizational prerequisites for successful implementation.” | Thank you for this constructive comment. The aim is reformulated explicit addressing identification of organizational prerequisites in order to increase the usage of video consultations:  *This study aims to explore outpatient clinic nurses’ experiences with video consultations and identify the organisational prerequisites they consider important for successful implementation of video consultations in outpatient clinics.* |
| Reviewer #1 – Methodology and Theoretical Framework  The justification for combining hermeneutics with Braun & Clarke’s thematic analysis needs clarification. How are hermeneutic interpretive principles integrated beyond general reflexivity? | Thank you for this comment. We have clarified combination of hermeneutic approach and the use of Braun and Clarke. Further we have integrated the interpretive principles as we have highlighted, how we worked with data in relation to meaning, context and understanding:  *The hermeneutic approach was used to facilitate a deeper understanding of the interview text by alternating between parts of the data and the dataset as a whole, interpreting the findings by continually questioning their meaning (Polit & Beck, 2017). The analysis followed the six phases of thematic analysis recommended by Braun and Clarke (2006), as we wanted to work systematically with data while interpreting it in terms of meaning, context and understanding. Braun og Clarke offer a systematic structure for analysing qualitative data:* |
| Reviewer #1 – Methodology and Theoretical  Clarify sampling rationale beyond convenience (why 12 participants, why two clinics). | Thank you for this comment, we have specified the sampling rationale:  *Two focus group interviews were conducted, one in each location familiar to the participants to create a comfortable contextually relevant setting and to avoid transport time for the participants. Different locations also allowed the study to capture potential variations in local practices and perspectives across the outpatient clinic sites.*  (…)  The interview dates were chosen by the first and last author, and all nurses from the outpatient clinic working on the selected dates were invited to participate in the focus group interviews. In total 17 clinical nurses. The nurse managers ensured that information about the interviews was communicated, and that time was allocated within the outpatient nursing programs.  *All outpatient clinic nurses were invited to participate in the focus groups. None were excluded. The participants in each focus group were all female nurses working in cardiological clinical practice and knew each other from their work (Table 2).* |
| Reviewer #1 – Methodology and Theoretical  Provide details on inclusion/exclusion criteria for participants. | Thank you for this comment, please see the revised wording above:  *All outpatient clinic nurses were invited to participate in the focus groups. None were excluded.* |
| Reviewer #1 – Methodology and Theoretical  Specify the ethical approvals in full alignment with Danish regulations and the institutional ethics process. The statement "did not require approval" should be accompanied by a brief justification and journal number (already present—retain that clarity). | Thank you for this comment. We have clarified the need for ethical approvals:  *The study did not require approval from Danish research ethics committees (Journal No. F-23075094), since the study does not involve experiments on live-born human individuals, human gametes, experiments with drugs, testing of medical devices or similar procedures*. |
| Reviewer #1 – Methodology and Theoretical  Explicitly cite the use of COREQ checklist in the methods section rather than only mentioning adherence. | Thank you for this comment. We have explicated the purpose of using COREQ:  *To ensure transparency and completeness in reporting, the study followed the Consolidated Criteria for Reporting Qualitative Research (COREQ) checklist. The checklist guided the study throughout its development, execution, and reporting phases (Tong et al., 2007).* |
| Reviewer #1 – Data collection  Include recruitment steps (who invited participants, selection criteria) and how voluntary participation was ensured. | Thank you for this constructive comment.  The section about choice of date for interview and invitation is incorporated in Sampling and recruitment instead of ethical consideration. Further, in continuation to the above answers, the recruitment process is elaborated:  *The interview dates were chosen by the first and last author, and all the nurses in the outpatient clinic working on the selected dates were invited to participate in the focus group interviews. In total 17 clinical nurses. The nurse managers ensured that information about the interviews was communicated, and that time was allocated within the outpatient nursing programs.*  *All outpatient clinic nurses were invited to participate in the focus groups. None were excluded. The participants in each focus group were all female nurses working in cardiological clinical practice and knew each other from their daily work. Although the invited outpatient clinic nurses were relatively homogeneous, variations in participants’ digital experiences as well as attitudes and preferences were assumed variated. Exploring these differences was considered important to support a relevant subsequent implementation. Four invited nurses cancelled due to having scheduled time off, and one due to being too busy on the interview day. (Table 2).* |
| Reviewer #1 – Data collection  Briefly describe the environment (online/in person, language used) and how confidentiality was maintained during group discussions. | *Thank you for this constructive comment. We have added a section describing the interview environment:*  *Physical interviews were conducted on the hospital wards where the respective groups of outpatient nurses were employed. The interviews were conducted in the afternoon, at a time when most participants had no scheduled patient consultations. Conducting the interviews physically enabled attention to body language and interaction patterns while supporting open discussions and nuanced exchanges within the relatively homogeneous group. The interviews were conducted in the participants' native language (Danish).*  Further, we have elaborated how confidentiality was maintained:  *To support a safe and confidential discussion environment, the interviews were initiated with a short informal chat followed by a clear and transparent framing of the study aim before diving into the topic. The intended use of the data and the procedures related to anonymity and confidentiality were clarified. Verbal and nonverbal facilitation techniques were used throughout the interviews to acknowledge and accommodate difficult or conflicting emotions and viewpoints. The moderators applied affirmative communication and active listening strategies and deliberately avoided interrupting participants. Further progressively focusing the participants’ attention on the topics of most significant interest (Krueger, 2006).* |
| Reviewer #1 – Data collection  Indicate whether field notes or memos were kept and how they informed the analysis. | Thank you for this comment. For your information, data consisted of the transcribed interview, we didn’t use field notes or memos. |
| Reviewer #1 – Data analysis  Describe how disagreement among coders was resolved. | Thank you for this comment. The section has been expanded in relation to the authors' collaboration in the analysis process.  *Researcher triangulation was conducted by the first and last author to validate the analysis in phase 4-5. Coded data were used as a basis for reflective discussions between the first and last author about content and meaning, and consensus was reached through these discussions to achieve a nuanced understanding of the data.*  *The themes were not understood as emerging directly from the data but were actively generated through the researchers’ interpretative engagement with patterns of shared meaning across the coded dataset.* |
| Reviewer #1 – Data analysis  Explain the link between the initial codes and the final 3 themes (the appendix helps but could use a clearer narrative in the results). | Thank you for this constructive comment. The link between the initial codes and the final 3 themes has been made explicit in the beginning of the result section:  *Three themes were developed through the interpretative process based on 12 codes: 1) Video consultations must be used at the right time, was developed from codes describing how nurses assessed the appropriateness and potential value of video consultations in specific clinical situations, including relative involvement, cross-sectoral collaboration, opportunities for new observations, and nurses’ professional judgement in selecting consultation types 2) Nursing care must remain as a relational practice was developed from codes reflecting concerns about how video consultations influenced relational and professional aspects of nursing practice. These included concerns about the patient relationship, reduced work satisfaction, the complexity of nursing practice, and the perceived risk that video consultations could devalue nursing competencies. 3) Missing initiatives for enabling nurses to use video consultations. was developed from codes related to organisational, technical, and practical barriers to implementation. These included the perceived flexibility of telephone consultations compared with video consultations, lack of organisational and technical support, and the need for knowledge and competencies in facilitating video consultations. These themes are presented in Table 3, supported by illustrative, representative, and rich quotations.* |
| Reviewer #1 – Data analysis  Consider adding brief justification for thematic saturation (how was it determined?). | Thank you for this constructive comment. We have elaborated on this:  *Data collection was considered sufficient and data saturation achieved, when the interviews provided rich and nuanced insight relevant to the research aim and to the context of video consultation implementation.* |
| Reviewer #1 – Data analysis  Add verbatim quotations’ translation procedure (if Danish; who translated; checked for accuracy). | Thank you for this comment. We have elaborated this:  *For dissemination purposes, the transcribed data used to support the result were translated by the first author. Translation accuracy was verified by the first and last author.* |
| Reviewer #1 - Results  Greater analytical depth could be added: move beyond description to subtle interpretation — e.g., what do these findings imply about nurses' professional identity or care ethics? | Thank you for this constructive comment. We have expanded the presentation of the results by adding more analytical dept e.g.:  *The participants expressed ambivalence regarding the justification for video consultations compared to physical consultations, framing them primarily through an efficiency-driven logic rather than clinical or relational rationale: (quote, P1)*  *The quote illustrates how video consultations were received as embedded in a broader organisational rationality of optimisation and time-efficiency, which was not necessarily aligned with nurses’ professional judgement of what constituted “good care”.*  and  *The nurse’s professional identity seems deeply grounded in embodied, relational and ethically orientated care. A participant described feeling that video consultations diminished the value of her education and clinical experience by undercutting her professional competencies:* |
| Reviewer #1 - Results  Consider summarizing each theme at the end of its section with a concise interpretive statement connecting it to the study aim. | All themes are shortly summarized in the end of each section:  1: *The findings indicate that increased use of video consultations depends on aligning technological options with nurses’ clinical judgement, patient preferences, and organisational structures that support flexible and coordinated care delivery.*  *2: The findings indicate that ambivalence towards video consultations reflects a tension between efficiency-driven organisational logics and nurses’ relational and embodied understanding of “good care”, and highlights nurse-identified prerequisites for increased use, aligning digital solutions with professional identity clear communication and organisational support.*  *3: The findings suggest that increased use of video depends on aligning managerial expectations with clinical realities particularly by addressing time constraints, limited implementation support, and the fit between digital workflows and everyday nursing practise.* |
| Reviewer #1 - Results  Check the balance of direct quotes; some are lengthy and could be trimmed while preserving richness. | Thank you for this comment. We have trimmed some of the quotes, e.g.:  *‘~~I see their faces on the screen, and~~ I don’t always remember what they look like if I speak with them on the phone. ~~So, I see their faces, and I enter their living room for better or worse.~~ (…) You can get some information about their daily lives in a way. (…) Yesterday, one was smoking while we were having the consultation; she only took one drag of the cigarette, but at least it was enough for me to remember asking her to consider stopping smoking’. (2)* |
| Reviewer #1 - Results  Numbered participant codes are fine but should be presented consistently (e.g., “(P1)” rather than “Participant 1”). | All participants are revised from e.g. Participant 1 to P1 |
| Reviewer #1 - Discussion  The discussion occasionally repeats findings rather than interpreting them further. Focus on why nurses react as they do, not just that they react. | Thank you for this constructive comment. We have elaborated on that through the discussion section, e.g.:  *The nurses acknowledged their role in initiating video consultations, and actively decided which patients to offer video consultations, based on what they deemed best for their patients, potentially positioning patients in a passive role*  And  *The nurses found achieving this presence challenging without physical interaction. In response, they often prioritise physical consultations for first meetings and consciously select consultation formats that they perceive as better suited to relational assessment.*  And  *The participants pointed out the need to develop their technological skills before offering video consultations. In response, nurses experiment with the technology in practice, test video links with patients, and gradually integrate or discontinue use depending on perceived usability and success.* |
| Reviewer #1 - Discussion  Could better discuss the organizational implications for nursing leadership and digital implementation strategy. | Thank you for this constructive comment. The discussion section about the finding *Missing initiatives for enabling nurses to use video consultations’* is revised to include more explicit implications for nursing leadership. Also, a more separated description of the nursing leadership responsibility for the whole process of digitalising health care, e.g.:  *The finding of ‘Missing initiatives for enabling nurses to use video consultations’ revealed that organisational leadership, prioritisation and strategic direction are crucial in successful digital implementation. Nursing leadership plays a central role in translating digital health technologies into sustainable clinical practice by ensuring alignment between organisational goals, resources and workflows. Managerial encouragement, structured training, and the provision of resources were found to be imperative for enabling nurses to employ video consultations (Kats & Shmueli, 2024).* |
| Reviewer #1 - Discussion  Consider grouping discussion under subheadings that reflect the three main themes — improves readability and alignment. | Thank you for this great comment, we have decided to keep the discussion in its current form. |
| Reviewer #1 - Discussion  Limit quotations in the discussion—focus on synthesis. | Thank you for this comment. The discussion section does only contain theme headings. |
| Reviewer #1 – Strengths and limitations  Add a short reflection on limitations of generalizability given the Danish context and small sample size. | Thank you for this constructive comment. We have added the following section on strength and limitation in relation to context and sample size:  *Finally, the study’s transferability may be limited by the relatively small sample size in a Danish context and the inclusion of participants from only one department, albeit across two sites. Although the findings provide valuable contextual insights, they may not be fully generalisable to other settings.* |
| Reviewer #1 – Strengths and limitations  Balanced coverage but could structure more clearly into “Strengths” and “Limitations.” | Thank you for this comment. We have divided the section in strength and limitations to clarify the content. |
| Reviewer #1 – Strengths and limitations  Mention possible response bias due to participants’ shared workplace and existing relationships. | Thank you for this comment. Considerations regarding potential response bias are included:  *Possible response bias may exist due to the shared workplace and pre-existing relationships between the interviewers and participants. However, efforts were made to reduce this risk by thoroughly informing participants at the beginning of the interviews about anonymity and confidentiality, and by emphasizing that the interviews would contribute important knowledge to the planning and organising of the further implementation process.* |
| Reviewer #1 – Strengths and limitations  Add comment on data saturation confirmation process. | We have elaborated the process of confirmation data saturation:  *Coding accuracy and reliability were measured by the research team. Further, the research team discussed coding for sufficient data saturation.* |
| Reviewer #1 – Strengths and limitations  Clear and constructive. Could more explicitly tie recommendations to education, policy, and future longitudinal or intervention studies on nurse adoption of telehealth. | Thank you for this constructive comment. The strengths and limitations section has been revised to ensure a clearer structural separation and a more balanced content.  Recommendations for further research is elaborated in the manuscript:  *Future research should focus on practice-oriented approaches to implementation. Comparative studies examining differences in patient outcomes, communication quality, and workflow efficiency between video, telephone, and physical consultations would provide valuable insights into the most appropriate use of each consultation format. In addition, interventional studies evaluating the effect of structured nurse training programs, implementation strategies, or technological support initiatives could help identify effective methods for strengthening staff competencies and confidence in video consultations. Multi-center studies across outpatient clinics and healthcare settings are also recommended to explore organisational differences.* |
| Reviewer #1 – Strengths and limitations  Suggest testing implementation frameworks (e.g., CFIR or NASSS) in future work. | Thank you for this comment. We will consider that in future work. |
| Reviewer #1 - Conclusion  Consider emphasizing practical implications for hospital administrators and educators. | The conclusion has been updated to provide clearer implications for practice and a stronger organizational perspective.  The section below  *From a policy and organisational perspective, the findings highlight the need for sustained investment in training, clear implementation strategies, and leadership that actively aligns digital initiatives with clinical realities and professional nursing values.*  Further,  *Overall, sustainable digital transformation in outpatient care depends on ensuring that technological development supports rather than undermines the relational and ethical foundations of nursing practice, which requires active support, reflection, and prioritisation from both leaders and educators.* |
| Reviewer #1 - Conclusion  The conclusion could end with a stronger statement connecting relational nursing ethics to sustainable digital transformation in healthcare. | Thank you for this constrictive comment. The conclusion is summarised with the following closing:  *Overall, the findings suggest that increased use of video consultations depends on organisational and managerial support that aligns digital solutions with nurses’ relational and ethical understanding of nursing care.* |
| Reviewer #1 – References  Formatting should be as per journal guidelines. | Thank you for this comment. We have revised the references formatting to journal guidelines. |
| Reviewer #1 - References  Ensure uniform use of ampersands (“&”) and en-dashes for page ranges. | Thanks for the comments. we have changed the reference system to Vancouver, and it should now be corrected |
| Reviewer #1 - References  Verify correct DOIs formatting and spacing (some inconsistencies noted). | Thank you for the comment. See the above answer. |
| Reviewer #1 – Tables and appendix  Table 1 (interview guide) and Table 2 (participant characteristics) are clear and helpful. | Thank you for this comment |
| Reviewer #1 – Tables and appendix  Table 3 effectively demonstrates coding traceability but could include one more example per theme for greater transparency. | Thank you for this comment. For the sake of clarity, we have left content of the table as it is. |
| Reviewer #1 – Tables and appendix  Ensure tables conform to journal typesetting format. | Thank you for this comment. We have entered content in separate cells. |
| Reviewer #2:  The title lacks methodological and contextual specificity, reducing clarity and discoverability, so include the study design and setting (e.g., qualitative focus group study in Danish cardiology outpatient clinics) and correct the grammatical form to “Nurses’ perspectives.” | The title ‘*Exploring Nurses’ Perspectives on Video Consultations and the Nurse–Patient Relationship’* is reformulated to:  *“Exploring Nurses’ perspectives on Video Consultations and the nurse–patient relationship: Findings from qualitative focus group interviews in a Danish cardiology outpatient setting”* |
| Reviewer #2  The abstract is descriptive but lacks precision, methodological clarity, and a clearly stated research gap, so explicitly state the gap, refine the aim, include specific methodological details and richer results, and end with a concise, actionable conclusion. | The abstract has been reformulated with clarity in research gap, aim, methodological details and richer results.  See also comments to reviewer, who have addressed the same issues. |
| Reviewer #2  The introduction presents fragmented literature without a clearly articulated gap or strong narrative flow, so strengthen the problem statement with evidence, synthesize literature critically, define key terms, and conclude with a clear research gap and research question. The aim is repetitive and lacks precision in defining the study focus, so refine it into a concise and clearly defined statement specifying the dimensions of nurses’ experiences being explored. | Thank you for this constructive and detailed feedback. We have revised the introduction to improve the overall flow reducing fragmentation. Key concepts have been more clearly defined, and the section now concludes with a clearer representation of the research gap. In addition, the aim has been refined:  “*This study aims to explore outpatient clinic nurses’ experiences with video consultations and identify the organisational prerequisites they consider important for successful implementation of video consultations in outpatient clinics.”* |
| Reviewer #2  The rationale for using a hermeneutic approach is insufficiently justified and operationalized, so clearly justify the methodological choice and explain how hermeneutic principles guided the analysis in practice. | Thank you for this constructive comment. We have clarified the rationale for using the hermeneutic approach:  *The hermeneutic approach recognises that understanding is always situated and influenced by the researchers’ pre-existing knowledge and understanding, which is made explicit and reflected upon throughout the analytical process. Interpretation of the findings, is developed through continuous movement between parts of the data and the whole, allowing meaning to evolve during analysis (Polit & Beck, 2017).* |
| Reviewer #2  The sampling strategy lacks justification and does not address potential biases or limitations, so justify the sample size using qualitative principles, clarify inclusion criteria, and discuss limitations such as homogeneity and selection bias.  The data collection process lacks sufficient detail to ensure transparency and reproducibility, so provide information on interview setting, pilot testing, probing techniques, and researcher influence on data collection.  There is inconsistency and insufficient clarity regarding ethical approval and procedures, so ensure consistency in ethical statements and clearly justify exemption while detailing consent, confidentiality, and data protection measures. | Thank you for this constructive comment. We have elaborated the whole section about data collection to clarify the process to ensure transparency:  *The interview guide was created based on a systematic literature review, clinical experiences, and qualifications of the first and last authors. Although the interview guide was not formally pilot tested, it was thoroughly discussed and refined among the first and last author prior the interviews.*  See also comments to reviewer, who have addressed the same issues. |
| Reviewer #2  The discussion is repetitive and lacks critical engagement with existing literature and theoretical contribution, so focus on interpreting key findings, critically compare with prior studies, highlight theoretical implications, and avoid repeating results. | Thank you for this constructive comment. Initially, in the discussion section, we have highlighted key findings:  *The findings provided a nuanced understanding of the factors that can affect the use of video consultations, suggesting that their limited integration is not only a question of technology, but equally a matter of contextual fit, professional judgment, and organisational support. When these factors are not adequately addressed, it seems they contribute to the continued limited use of video consultations in outpatient clinic settings.*  Further, we have highlighted theoretical implications, e.g.:  *Video consultations convenience aligns with findings from another study, which reported video consultations as significant time and cost savers for patients (Donaghy et al., 2019). Together, the results point to a need to rethink the decision-making process, which could benefit from taking a more patient-centered approach and shared decision-making rather than what the nurse assesses is best in the situation,*  *The nurses in this study expressed concerns about fulfilling their ethical and moral responsibilities through video consultations. This finding has important theoretical implications, as relational nursing theory does not include digitality, its limitations and conditions, which challenges the desired digital implementation in healthcare.* |
| Reviewer #2  The strengths and limitations section is unbalanced and does not sufficiently acknowledge key study limitations, so clearly separate these elements and critically discuss issues such as small sample size, lack of diversity, and contextual constraints. | Thank you for this constructive comment. the strengths and limitations section has been revised to ensure a clearer structural separation and a more balanced content. |
| Reviewer #2  The recommendations for further research are broad and lack specificity, so provide focused and actionable suggestions such as comparative, interventional, or multi-center studies. | Thank you for this constructive comment. We have elaborated the recommendations for further research with the following section:  Future research should focus on practice-oriented approaches to implementation. Comparative studies examining differences in patient outcomes, communication quality, and workflow efficiency between video, telephone, and physical consultations would provide valuable insights into the most appropriate use of each consultation format. In addition, interventional studies evaluating the effect of structured nurse training programs, implementation strategies, or technological support initiatives could help identify effective methods for strengthening staff competencies and confidence in video consultations. Multi-center studies across outpatient clinics and healthcare settings are also recommended to explore organizational differences. |
| Reviewer #2  The conclusion is lengthy and reiterates earlier sections without delivering a strong final message, so make it concise, highlight key findings, and clearly state practical and policy implications. | Thank you for this constructive comment. We have reformulated the conclusion and added clear points about the importance for practice and organizational perspective. Further, a clear final message:  Overall, sustainable digital transformation in outpatient care depends on ensuring that technological development supports rather than undermines the relational and ethical foundations of nursing practice, which requires active support, reflection, and prioritization from both leaders and educators. |
| Reviewer #2  The tables are complex and not optimally structured for clarity and interpretation, so simplify formatting, ensure consistency, and improve alignment between codes, themes, and supporting data. | Thank you for this comment. For the sake of clarity, we have left content of the table as it is. Further, We have entered content in separate cells. |
| Reviewer #2  The analysis description is overly generic and does not clearly reflect what was actually done, so clarify coding procedures, analytic decisions, researcher roles, and how rigor (credibility and dependability) was ensured | Thank you for this comment. We have elaborated the analysis section to clarify what was actually done:  *First author completed phase 1-3.*  *Phase 4: Reviewing themes required refining the themes through critical reviewing. First, we considered whether the codes formed a coherent pattern and adequately captured the perspectives within the coded data. Second, reading alternating sections of the text generated new understandings of the individual sections and the text as a whole. The refinement process continued until no substantial new insights were added.*  *Phase 5: Defining and naming themes involved identifying the ‘essence’ of each theme and determining what aspect of the data each theme captured.*  *Researcher triangulation was conducted by the first and last author to validate the analysis in phase 4-5. Coded data were used as a basis for reflective discussions between the first and last author about content and meaning, and consensus was reached through these discussions to achieve a nuanced understanding of the data. The themes were not understood as emerging directly from the data but were actively generated through the researchers’ interpretative engagement with patterns of shared meaning across the coded dataset.* |
| Reviewer #2  The results are largely descriptive with limited analytical depth and lack subtheme structure, so introduce subthemes, enhance interpretive analysis, reduce excessive quotations, and clearly link codes to themes. | Thank you for this constructive comment.  We have elaborated the result section to present more analytical depth, e.g.:  *A common view was an overall concern about the increasing use of technological solutions in outpatient clinic treatment and care activities., which illustrates an underlying tension between technological expansion and professional discretion in clinical decision-making.*  And  *The participants expressed that the first meeting with a patient should preferably be a physical consultation because it establishes the relationship. This reflects an implicit hierarchy of consultation forms, where physical presence is constructed as foundational for relationship-building.*  *And*  *The organisation of the outpatient clinics and scheduling of patients sometimes acted as barriers to video consultations because only telephone consultations could be flexibly added as an extra option. This points to a misalignment between technological possibilities and infrastructural realities* |
