# Supplementary material for: Exploring nurses’ perspectives on video consultations and the nurse–patient relationship: Findings from qualitative focus group interviews in a Danish cardiology outpatient setting
Source: PLoS One. 2026 Aug 11;21(8):e0339752. doi: 10.1371/journal.pone.0339752 (PMC13460611; doi:10.1371/journal.pone.0339752)
Supplement: Peer review history

# Peer review: Decision Letter 0

**DOI:** 10.1371/journal.pone.0339752.r001

**Authors:** Nagendra Rai

27 Apr 2026

Dear Dr. Groth,

Thank you for submitting your manuscript to PLOS ONE. After careful consideration, we feel that it has merit but does not fully meet PLOS ONE’s publication criteria as it currently stands. Therefore, we invite you to submit a revised version of the manuscript that addresses the points raised during the review process.

Please submit your revised manuscript by Jun 11 2026 11:59PM. If you will need more time than this to complete your revisions, please reply to this message or contact the journal office at plosone@plos.org. When you're ready to submit your revision, log on to https://www.editorialmanager.com/pone/ and select the 'Submissions Needing Revision' folder to locate your manuscript file.

- A letter that responds to each point raised by the academic editor and reviewer(s). You should upload this letter as a separate file labeled 'Response to Reviewers'.
- A marked-up copy of your manuscript that highlights changes made to the original version. You should upload this as a separate file labeled 'Revised Manuscript with Track Changes'.
- An unmarked version of your revised paper without tracked changes. You should upload this as a separate file labeled 'Manuscript'.

If you would like to make changes to your financial disclosure, please include your updated statement in your cover letter. Guidelines for resubmitting your figure files are available below the reviewer comments at the end of this letter.

If applicable, we recommend that you deposit your laboratory protocols in protocols.io to enhance the reproducibility of your results. Protocols.io assigns your protocol its own identifier (DOI) so that it can be cited independently in the future. For instructions see: https://journals.plos.org/plosone/s/submission-guidelines#loc-laboratory-protocols. Additionally, PLOS ONE offers an option for publishing peer-reviewed Lab Protocol articles, which describe protocols hosted on protocols.io. Read more information on sharing protocols at https://plos.org/protocols?utm_medium=editorial-email&utm_source=authorletters&utm_campaign=protocols.

As the corresponding author, your ORCID iD is verified in the submission system and will appear in the published article. PLOS supports the use of ORCID, and we encourage all coauthors to register for an ORCID iD and use it as well. Please encourage your coauthors to verify their ORCID iD within the submission system before final acceptance, as unverified ORCID iDs will not appear in the published article. *Only*  the individual author can complete the verification step; PLOS staff *cannot*  verify ORCID iDs on behalf of authors.

We look forward to receiving your revised manuscript.

Kind regards,

Nagendra Kumar Rai, Ph.D

Guest Editor

PLOS One

Journal Requirements:

When submitting your revision, we need you to address these additional requirements.

1.Please ensure that your manuscript meets PLOS ONE's style requirements, including those for file naming. The PLOS ONE style templates can be found at

https://journals.plos.org/plosone/s/file?id=wjVg/PLOSOne_formatting_sample_main_body.pdf and

https://journals.plos.org/plosone/s/file?id=ba62/PLOSOne_formatting_sample_title_authors_affiliations.pdf

2. Thank you for stating the following financial disclosure:

“Lundbeck Foundation”

Please state what role the funders took in the study.  If the funders had no role, please state: "The funders had no role in study design, data collection and analysis, decision to publish, or preparation of the manuscript."

If this statement is not correct you must amend it as needed.

Please include this amended Role of Funder statement in your cover letter; we will change the online submission form on your behalf.

3. We note that the grant information you provided in the ‘Funding Information’ and ‘Financial Disclosure’ sections do not match.

When you resubmit, please ensure that you provide the correct grant numbers for the awards you received for your study in the ‘Funding Information’ section.

4. We note that your Data Availability Statement is currently as follows: All relevant data are within the manuscript and in Supporting Information files.

Please confirm at this time whether or not your submission contains all raw data required to replicate the results of your study. Authors must share the “minimal data set” for their submission. PLOS defines the minimal data set to consist of the data required to replicate all study findings reported in the article, as well as related metadata and methods (https://journals.plos.org/plosone/s/data-availability#loc-minimal-data-set-definition).

For example, authors should submit the following data:

- The values behind the means, standard deviations and other measures reported;

- The values used to build graphs;

- The points extracted from images for analysis.

Authors do not need to submit their entire data set if only a portion of the data was used in the reported study.

If your submission does not contain these data, please either upload them as Supporting Information files or deposit them to a stable, public repository and provide us with the relevant URLs, DOIs, or accession numbers. For a list of recommended repositories, please see https://journals.plos.org/plosone/s/recommended-repositories.

If there are ethical or legal restrictions on sharing a de-identified data set, please explain them in detail (e.g., data contain potentially sensitive information, data are owned by a third-party organization, etc.) and who has imposed them (e.g., an ethics committee). Please also provide contact information for a data access committee, ethics committee, or other institutional body to which data requests may be sent. If data are owned by a third party, please indicate how others may request data access.

5. We note that the main manuscript consists of interview transcripts. Can you please confirm that all participants gave consent for interview transcript to be published?

If they DID provide consent for these transcripts to be published, please also confirm that the transcripts do not contain any potentially identifying information (or let us know if the participants consented to having their personal details published and made publicly available). We consider the following details to be identifying information:

- Names, nicknames, and initials

- Age more specific than round numbers

- GPS coordinates, physical addresses, IP addresses, email addresses

- Information in small sample sizes (e.g. 40 students from X class in X year at X university)

- Specific dates (e.g. visit dates, interview dates)

- ID numbers

Or, if the participants DID NOT provide consent for these transcripts to be published:

- Provide a de-identified version of the data or excerpts of interview responses

- Provide information regarding how these transcripts can be accessed by researchers who meet the criteria for access to confidential data, including:

a) the grounds for restriction

b) the name of the ethics committee, Institutional Review Board, or third-party organization that is imposing sharing restrictions on the data

c) a non-author, institutional point of contact that is able to field data access queries, in the interest of maintaining long-term data accessibility.

d) Any relevant data set names, URLs, DOIs, etc. that an independent researcher would need in order to request your minimal data set.

For further information on sharing data that contains sensitive participant information, please see: https://journals.plos.org/plosone/s/data-availability#loc-human-research-participant-data-and-other-sensitive-data

If there are ethical, legal, or third-party restrictions upon your dataset, you must provide all of the following details (https://journals.plos.org/plosone/s/data-availability#loc-acceptable-data-access-restrictions):

1. A complete description of the dataset

2. The nature of the restrictions upon the data (ethical, legal, or owned by a third party) and the reasoning behind them

3. The full name of the body imposing the restrictions upon your dataset (ethics committee, institution, data access committee, etc)

4. If the data are owned by a third party, confirmation of whether the authors received any special privileges in accessing the data that other researchers would not have

5. Direct, non-author contact information (preferably email) for the body imposing the restrictions upon the data, to which data access requests can be sent

6. If the reviewer comments include a recommendation to cite specific previously published works, please review and evaluate these publications to determine whether they are relevant and should be cited. There is no requirement to cite these works unless the editor has indicated otherwise.

Additional Editor Comments:

Dear Authors,

Thank you for submitting your manuscript. The reviewers find the topic timely and the qualitative approach appropriate, but they raise several concerns that need to be addressed before further consideration.

Key issues include:

Insufficient clarity in the research gap, aim, and overall focus

Limited methodological transparency (sampling, data collection, analysis) and incomplete alignment with COREQ standards

Predominantly descriptive results with limited interpretive depth

Repetition and lack of precision in parts of the abstract, introduction, and discussion

Please revise the manuscript to strengthen methodological rigor, enhance analytical depth, and improve clarity and structure throughout.

Decision: Major Revision

[Note: HTML markup is below. Please do not edit.]

Reviewers' comments:

Reviewer's Responses to Questions

**Comments to the Author**

1. Is the manuscript technically sound, and do the data support the conclusions?

Reviewer #1: Partly

Reviewer #2: Yes

**********

2. Has the statistical analysis been performed appropriately and rigorously?

Reviewer #1: Yes

Reviewer #2: Yes

**********

3. Have the authors made all data underlying the findings in their manuscript fully available?

The PLOS Data policy

Reviewer #1: Yes

Reviewer #2: Yes

**********

4. Is the manuscript presented in an intelligible fashion and written in standard English?

Reviewer #1: Yes

Reviewer #2: Yes

**********

Reviewer #1: General Assessment

This qualitative study addresses a timely and relevant topic — nurses’ perspectives on video consultations and their influence on the nurse–patient relationship in outpatient cardiology settings. The focus on implementation barriers and relational aspects adds valuable insight to healthcare digitization efforts. The manuscript is well-structured, coherent, and grounded in established qualitative methodology (hermeneutic and thematic analysis).

However, revisions are recommended to strengthen clarity, ensure methodological transparency, align with reporting standards (e.g., COREQ), and enhance the discussion’s critical engagement with existing literature.

Title and Abstract

Strengths:

• The title accurately reflects the study’s focus and design.

• The abstract clearly outlines the aim, methodology, results, and conclusions.

Areas for Improvement:

• The “Materials and methods” section in the abstract could more succinctly indicate the theoretical approach and sampling rationale.

• Include precise information on the analysis method (e.g., “thematic analysis guided by Braun & Clarke”).

• Avoid repetition of aims; integrate the second sentence into a single cohesive aim statement.

• Mention the number of focus groups and participants directly in the abstract for transparency.

Keywords

Comments:

• The selected keywords are appropriate; consider adding “telehealth” or “telemedicine” to align with global terminology and indexing conventions.

Introduction

Strengths:

• Provides clear rationale linking global telehealth trends with the Danish context.

• Demonstrates solid citation of relevant WHO and current literature.

Areas for Improvement:

• The introduction is rich but somewhat dense. Consider tightening for flow and removing minor redundancies (e.g., overlapping statements about the pandemic’s influence).

• The final paragraph should more clearly articulate the gap in existing knowledge leading to this study (e.g., “Few studies have explored nurses’ experiential and relational perspectives in cardiology outpatient contexts…”).

• Clarify the conceptual grounding: Is the study primarily exploring perceptions and relational care or implementation barriers? Both are present but could be better integrated.

Aim and Objectives

Comments:

• The aim is clearly stated but repeated in several sections (abstract, methods, and introduction). Condense to one consistent phrasing.

• Consider adding a secondary objective explicitly addressing “identification of organizational prerequisites for successful implementation.”

Methodology and Theoretical Framework

Strengths:

• Sound use of a hermeneutic qualitative approach and thematic analysis.

• Acknowledgement of the researchers’ preunderstanding interview is excellent for reflexivity.

Areas for Improvement:

• The justification for combining hermeneutics with Braun & Clarke’s thematic analysis needs clarification. How are hermeneutic interpretive principles integrated beyond general reflexivity?

• Clarify sampling rationale beyond convenience (why 12 participants, why two clinics).

• Provide details on inclusion/exclusion criteria for participants.

• Specify the ethical approvals in full alignment with Danish regulations and the institutional ethics process. The statement "did not require approval" should be accompanied by a brief justification and journal number (already present—retain that clarity).

• Explicitly cite the use of COREQ checklist in the methods section rather than only mentioning adherence.

Data Collection

Strengths:

• Well-described focus group structure, with justification for group size and facilitation strategy.

• Inclusion of an interview guide table is helpful.

Areas for Improvement:

• Include recruitment steps (who invited participants, selection criteria) and how voluntary participation was ensured.

• Briefly describe the environment (online/in person, language used) and how confidentiality was maintained during group discussions.

• Indicate whether field notes or memos were kept and how they informed the analysis.

Data Analysis

Strengths:

• Analysis procedure follows Braun & Clarke’s six phases, described in detail.

• Researcher triangulation and peer debriefing enhance credibility.

Areas for Improvement:

• Describe how disagreement among coders was resolved.

• Explain the link between the initial codes and the final 3 themes (the appendix helps but could use a clearer narrative in the results).

• Consider adding brief justification for thematic saturation (how was it determined?).

• Add verbatim quotations’ translation procedure (if Danish; who translated; checked for accuracy).

Results

Strengths:

• Results are well organized around three themes supported by illustrative quotations.

• Provides a rich description of nurses’ perspectives with both consensus and variation among participants.

Areas for Improvement:

• Greater analytical depth could be added: move beyond description to subtle interpretation — e.g., what do these findings imply about nurses' professional identity or care ethics?

• Consider summarizing each theme at the end of its section with a concise interpretive statement connecting it to the study aim.

• Check the balance of direct quotes; some are lengthy and could be trimmed while preserving richness.

• Numbered participant codes are fine but should be presented consistently (e.g., “(P1)” rather than “Participant 1”).

Discussion

Strengths:

• Comprehensive, well-cited, and theoretically grounded.

• Excellent integration of relational nursing philosophy (Løgstrup, Martinsen, Delmar).

• Nice synthesis with motivation theory (Deci & Ryan).

Areas for Improvement:

• The discussion occasionally repeats findings rather than interpreting them further. Focus on why nurses react as they do, not just that they react.

• Could better discuss the organizational implications for nursing leadership and digital implementation strategy.

• Consider grouping discussion under subheadings that reflect the three main themes — improves readability and alignment.

• Limit quotations in the discussion—focus on synthesis.

• Add a short reflection on limitations of generalizability given the Danish context and small sample size.

Strengths and Limitations

Comments:

• Balanced coverage, but could structure more clearly into “Strengths” and “Limitations.”

• Mention possible response bias due to participants’ shared workplace and existing relationships.

• Add comment on data saturation confirmation process.

Recommendations for Further Research

Comments:

• Clear and constructive. Could more explicitly tie recommendations to education, policy, and future longitudinal or intervention studies on nurse adoption of telehealth.

• Suggest testing implementation frameworks (e.g., CFIR or NASSS) in future work.

Conclusion

Strengths:

• Concise, grounded in results, and relevant.

Areas for Improvement:

• Consider emphasizing practical implications for hospital administrators and educators.

• The conclusion could end with a stronger statement connecting relational nursing ethics to sustainable digital transformation in healthcare.

References

Comments:

• Formatting should be as per journal guidelines.

• Ensure uniform use of ampersands (“&”) and en-dashes for page ranges.

• Verify correct DOIs formatting and spacing (some inconsistencies noted).

Tables and Appendix

Comments:

• Table 1 (interview guide) and Table 2 (participant characteristics) are clear and helpful.

• Table 3 effectively demonstrates coding traceability but could include one more example per theme for greater transparency.

• Ensure tables conform to journal typesetting format.

Data Availability, Ethics, and Funding Statements

Comments:

• Appropriate compliance statements are present and consistent with PLOS requirements.

Summary Recommendation

Decision: Major Revision

Rationale:

The manuscript presents a valuable and original qualitative contribution but requires refinement in structure, methodological detail, and interpretive depth before publication. Attention to these revisions will substantially enhance rigor, transparency, and scholarly impact.

Key Priority Actions:

1. Clarify integration of hermeneutic approach with thematic analysis.

2. Strengthen discussion to include interpretive analysis and practical implications.

3. Revise abstract and introduction for concise focus and clear identification of research gap.

Reviewer #2: The manuscript titled 'Exploring Nurses’ Perspectives on Video Consultations and the Nurse–Patient

Relationship' addresses a timely and relevant topic in healthcare, particularly the growing role of video consultations in nursing practice. The qualitative approach provides valuable insights into nurses’ perspectives, contributing meaningfully to the existing body of knowledge. The study is well-structured overall and demonstrates an effort to follow established qualitative reporting standards.

However, this manuscript still requires some major changes for its improvement. The authors are recommended to address the following comments and incorporate the same in the manuscript:

1. The title lacks methodological and contextual specificity, reducing clarity and discoverability, so include the study design and setting (e.g., qualitative focus group study in Danish cardiology outpatient clinics) and correct the grammatical form to “Nurses’ perspectives.”

2. The abstract is descriptive but lacks precision, methodological clarity, and a clearly stated research gap, so explicitly state the gap, refine the aim, include specific methodological details and richer results, and end with a concise, actionable conclusion.

3. The introduction presents fragmented literature without a clearly articulated gap or strong narrative flow, so strengthen the problem statement with evidence, synthesize literature critically, define key terms, and conclude with a clear research gap and research question.

4. The aim is repetitive and lacks precision in defining the study focus, so refine it into a concise and clearly defined statement specifying the dimensions of nurses’ experiences being explored.

5. The rationale for using a hermeneutic approach is insufficiently justified and operationalized, so clearly justify the methodological choice and explain how hermeneutic principles guided the analysis in practice.

6. The sampling strategy lacks justification and does not address potential biases or limitations, so justify the sample size using qualitative principles, clarify inclusion criteria, and discuss limitations such as homogeneity and selection bias.

7. The data collection process lacks sufficient detail to ensure transparency and reproducibility, so provide information on interview setting, pilot testing, probing techniques, and researcher influence on data collection.

8. There is inconsistency and insufficient clarity regarding ethical approval and procedures, so ensure consistency in ethical statements and clearly justify exemption while detailing consent, confidentiality, and data protection measures.

9. The analysis description is overly generic and does not clearly reflect what was actually done, so clarify coding procedures, analytic decisions, researcher roles, and how rigor (credibility and dependability) was ensured.

10. The results are largely descriptive with limited analytical depth and lack subtheme structure, so introduce subthemes, enhance interpretive analysis, reduce excessive quotations, and clearly link codes to themes.

11. The discussion is repetitive and lacks critical engagement with existing literature and theoretical contribution, so focus on interpreting key findings, critically compare with prior studies, highlight theoretical implications, and avoid repeating results.

12. The strengths and limitations section is unbalanced and does not sufficiently acknowledge key study limitations, so clearly separate these elements and critically discuss issues such as small sample size, lack of diversity, and contextual constraints.

13. The recommendations for further research are broad and lack specificity, so provide focused and actionable suggestions such as comparative, interventional, or multi-center studies.

14. The conclusion is lengthy and reiterates earlier sections without delivering a strong final message, so make it concise, highlight key findings, and clearly state practical and policy implications.

15. The tables are complex and not optimally structured for clarity and interpretation, so simplify formatting, ensure consistency, and improve alignment between codes, themes, and supporting data.

16. The manuscript contains grammatical inconsistencies and lacks stylistic precision, so revise for grammar, clarity, conciseness, and consistent academic tone and terminology.

17. Reporting lacks full transparency in alignment with qualitative reporting standards, so ensure complete adherence to COREQ guidelines including reflexivity, researcher roles, and data saturation.

18. Data transparency and reproducibility details are minimal and non-specific, so provide clearer data availability information and consider including supplementary materials such as coding frameworks and the full interview guide.

**********

what does this mean?). If published, this will include your full peer review and any attached files.

If you choose “no”, your identity will remain anonymous but your review may still be made public.

**Do you want your identity to be public for this peer review?**  For information about this choice, including consent withdrawal, please see our Privacy Policy

Reviewer #1: No

Reviewer #2: **Yes:**  Dr. Mohammad Fareed

**********

[NOTE: If reviewer comments were submitted as an attachment file, they will be attached to this email and accessible via the submission site. Please log into your account, locate the manuscript record, and check for the action link "View Attachments". If this link does not appear, there are no attachment files.]

To ensure your figures meet our technical requirements, please review our figure guidelines: https://journals.plos.org/plosone/s/figures

You may also use PLOS’s free figure tool, NAAS, to help you prepare publication quality figures: https://journals.plos.org/plosone/s/figures#loc-tools-for-figure-preparation.

NAAS will assess whether your figures meet our technical requirements by comparing each figure against our figure specifications.

# Author comment: Author response to Decision Letter 1

**DOI:** 10.1371/journal.pone.0339752.r002

16 Jun 2026

Response to reviewers

Journal Requirements:

Please ensure that your manuscript meets PLOS ONE's style requirements, including those for file naming We have revised the manuscript to meet style requirements.

Journal Requirements:

Please state what role the funders took in the study We have stated that the funders had no role in study design, data collection and analysis, decision to publish, or preparation of the manuscript

Journal Requirements:

When you resubmit, please ensure that you provide the correct grant numbers for the awards you received for your study in the ‘Funding Information’ section.

Thank you for this comment. There is no grand number for these funds.

Journal Requirements:

We note that your Data Availability Statement is currently as follows: All relevant data are within the manuscript and in Supporting Information files. Thank you for this comment, we will ensure that.

Journal Requirements:

We note that the main manuscript consists of interview transcripts. Can you please confirm that all participants gave consent for interview transcript to be published?

I confirm that all participants gave consent for interview transcript to be published. Please see the ethic section.

Additional Editor Comments:

Insufficient clarity in the research gap, aim, and overall focus Thank you for this comment. The introduction has been thoroughly updated, including the research gap, aim, and overall focus

Additional Editor Comments:

Limited methodological transparency (sampling, data collection, analysis) and incomplete alignment with COREQ standards Thank you for this comment, which has been elaborated. See the responses below to reviewer 1's comments.

COREQ checklist has been reviewed and updated.

Additional Editor Comments:

Predominantly descriptive results with limited interpretive depth Results have been elaborated with more interpretive depth. See the responses below to reviewer 1's comments

Additional Editor Comments:

Repetition and lack of precision in parts of the abstract, introduction, and discussion Thank you for this comment. We have reformulated the sections to be more clarified.

Se the answers below.

Reviewer #1 – Title and abstract

The “Materials and methods” section in the abstract could more succinctly indicate the theoretical approach and sampling rationale. Thank you for this suggestion. We have revised the “Materials and methods” section in the abstract to more succinctly clarify the theoretical approach underpinning the study and the rationale for the sampling strategy:

A hermeneutic qualitative exploratory approach was applied to explore and interpret

experiences and perceptions. Data were generated through two focus group interviews with 5 and 7 participating nurses in each group. The participants were recruited from two Danish cardiology outpatient clinics selected to ensure relevant clinical experience. Data were analysed using thematic analysis guided by Braun & Clarke.

Reviewer #1 - Title and abstract

Include precise information on the analysis method (e.g., “thematic analysis guided by Braun & Clarke”). We have revised the abstract to include a more precise description of the analysis method by explicitly stating that the data were analysed using thematic analysis guided by Braun & Clarke.

Reviewer #1 - Title and abstract

Avoid repetition of aims; integrate the second sentence into a single cohesive aim statement. Thank you for this constructive comment. We have revised the aim: This study aims to explore outpatient clinic nurses’ experiences with video consultations and to identify the organisational prerequisites they consider important to increase the usage of video consultations in outpatient clinics.

Reviewer #1 - Title and abstract

Mention the number of focus groups and participants directly in the abstract for transparency. Thank you for this helpful suggestion. We have revised the abstract to explicitly state the number of focus groups and participants to improve transparency and clarity:

Data were generated through two focus group interviews with 5 and 7 participating nurses in each group.

Reviewer #1 – Keywords

The selected keywords are appropriate; consider adding “telehealth” or “telemedicine” to align with global terminology and indexing conventions. Thank you for pointing the global terminology. We have added both “telehealth” or “telemedicine”.

Reviewer #1 - Introduction

The introduction is rich but somewhat dense. Consider tightening for flow and removing minor redundancies (e.g., overlapping statements about the pandemic’s influence). Thank you for this helpful comment. We have reformulated the introduction in general to improve overall flow and readability by tightening the text and removing repetitions, including overlapping statements.

‘Virtual healthcare’, ‘video consultations’ and ‘digital technologies’ were repeated many times, which has been attempted to be formulated more comprehensively and clearly. We have formulated it so that there is progression in flow: technology, challenge, implementation, perspectives and needs.

Reviewer #1 - Introduction

The final paragraph should more clearly articulate the gap in existing knowledge leading to this study (e.g., “Few studies have explored nurses’ experiential and relational perspectives in cardiology outpatient contexts…”). Thank you for this comment. We have revised the final paragraph to more clearly articulate the existing knowledge gap, particularly the limited research exploring nurses’ experiential and relational perspectives on video consultations in cardiology outpatient settings:

Although video consultations are increasingly used in healthcare, few studies have explored nurses’ experiential and relational perspectives in cardiology outpatient contexts. Existing research provides limited insight into how nurses experience the use of video consultations in clinical practise, including both the practical and organisational barriers to implementation and the ways video consultations may influence nursing care and patient relationships. Therefore, further research is needed to better understand nurses’ experiences with video consultations in cardiology outpatient clinics in order to identify and address barriers, support implementation in alignment with clinical priorities, and explore how video consultations may support or challenge relational aspects of nursing care for adult patients in outpatient clinic settings.

Reviewer #1 - Introduction

Clarify the conceptual grounding: Is the study primarily exploring perceptions and relational care or implementation barriers? Both are present but could be better integrated. Thank you for this constructive comment.

We have revised and clarified the link between video consultations and their importance of patient relationships and nursing care. Further the lack of research, which is essential to accommodate and handle the experienced barriers in implementation:

Existing research provides limited insight into how nurses experience the use of video consultations in clinical practice, including both the practical and organisational barriers to implementation and the ways video consultations may influence nursing care and patient relationships.

Reviewer #1 – Aim and objectives

The aim is clearly stated but repeated in several sections (abstract, methods, and introduction). Condense to one consistent phrasing. Thank you for this comment. The aim has been reformulated and ensured that it is consistent throughout the manuscript

Reviewer #1 – Aim and objectives

Consider adding a secondary objective explicitly addressing “identification of organizational prerequisites for successful implementation.” Thank you for this constructive comment. The aim is reformulated explicit addressing identification of organizational prerequisites in order to increase the usage of video consultations:

This study aims to explore outpatient clinic nurses’ experiences with video consultations and identify the organisational prerequisites they consider important for successful implementation of video consultations in outpatient clinics.

Reviewer #1 – Methodology and Theoretical Framework

The justification for combining hermeneutics with Braun & Clarke’s thematic analysis needs clarification. How are hermeneutic interpretive principles integrated beyond general reflexivity? Thank you for this comment. We have clarified combination of hermeneutic approach and the use of Braun and Clarke. Further we have integrated the interpretive principles as we have highlighted, how we worked with data in relation to meaning, context and understanding:

The hermeneutic approach was used to facilitate a deeper understanding of the interview text by alternating between parts of the data and the dataset as a whole, interpreting the findings by continually questioning their meaning (Polit & Beck, 2017). The analysis followed the six phases of thematic analysis recommended by Braun and Clarke (2006), as we wanted to work systematically with data while interpreting it in terms of meaning, context and understanding. Braun og Clarke offer a systematic structure for analysing qualitative data:

Reviewer #1 – Methodology and Theoretical

Clarify sampling rationale beyond convenience (why 12 participants, why two clinics). Thank you for this comment, we have specified the sampling rationale:

Two focus group interviews were conducted, one in each location familiar to the participants to create a comfortable contextually relevant setting and to avoid transport time for the participants. Different locations also allowed the study to capture potential variations in local practices and perspectives across the outpatient clinic sites.

(…)

The interview dates were chosen by the first and last author, and all nurses from the outpatient clinic working on the selected dates were invited to participate in the focus group interviews. In total 17 clinical nurses. The nurse managers ensured that information about the interviews was communicated, and that time was allocated within the outpatient nursing programs.

All outpatient clinic nurses were invited to participate in the focus groups. None were excluded. The participants in each focus group were all female nurses working in cardiological clinical practice and knew each other from their work (Table 2).

Reviewer #1 – Methodology and Theoretical

Provide details on inclusion/exclusion criteria for participants. Thank you for this comment, please see the revised wording above:

All outpatient clinic nurses were invited to participate in the focus groups. None were excluded.

Reviewer #1 – Methodology and Theoretical

Specify the ethical approvals in full alignment with Danish regulations and the institutional ethics process. The statement "did not require approval" should be accompanied by a brief justification and journal number (already present—retain that clarity). Thank you for this comment. We have clarified the need for ethical approvals:

The study did not require approval from Danish research ethics committees (Journal No. F-23075094), since the study does not involve experiments on live-born human individuals, human gametes, experiments with drugs, testing of medical devices or similar procedures.

Reviewer #1 – Methodology and Theoretical

Explicitly cite the use of COREQ checklist in the methods section rather than only mentioning adherence. Thank you for this comment. We have explicated the purpose of using COREQ:

To ensure transparency and completeness in reporting, the study followed the Consolidated Criteria for Reporting Qualitative Research (COREQ) checklist. The checklist guided the study throughout its development, execution, and reporting phases (Tong et al., 2007).

Reviewer #1 – Data collection

Include recruitment steps (who invited participants, selection criteria) and how voluntary participation was ensured. Thank you for this constructive comment.

The section about choice of date for interview and invitation is incorporated in Sampling and recruitment instead of ethical consideration. Further, in continuation to the above answers, the recruitment process is elaborated:

The interview dates were chosen by the first and last author, and all the nurses in the outpatient clinic working on the selected dates were invited to participate in the focus group interviews. In total 17 clinical nurses. The nurse managers ensured that information about the interviews was communicated, and that time was allocated within the outpatient nursing programs.

All outpatient clinic nurses were invited to participate in the focus groups. None were excluded. The participants in each focus group were all female nurses working in cardiological clinical practice and knew each other from their daily work. Although the invited outpatient clinic nurses were relatively homogeneous, variations in participants’ digital experiences as well as attitudes and preferences were assumed variated. Exploring these differences was considered important to support a relevant subsequent implementation. Four invited nurses cancelled due to having scheduled time off, and one due to being too busy on the interview day. (Table 2).

Reviewer #1 – Data collection

Briefly describe the environment (online/in person, language used) and how confidentiality was maintained during group discussions. Thank you for this constructive comment. We have added a section describing the interview environment:

Physical interviews were conducted on the hospital wards where the respective groups of outpatient nurses were employed. The interviews were conducted in the afternoon, at a time when most participants had no scheduled patient consultations. Conducting the interviews physically enabled attention to body language and interaction patterns while supporting open discussions and nuanced exchanges within the relatively homogeneous group. The interviews were conducted in the participants' native language (Danish).

Further, we have elaborated how confidentiality was maintained:

To support a safe and confidential discussion environment, the interviews were initiated with a short informal chat followed by a clear and transparent framing of the study aim before diving into the topic. The intended use of the data and the procedures related to anonymity and confidentiality were clarified. Verbal and nonverbal facilitation techniques were used throughout the interviews to acknowledge and accommodate difficult or conflicting emotions and viewpoints. The moderators applied affirmative communication and active listening strategies and deliberately avoided interrupting participants. Further progressively focusing the participants’ attention on the topics of most significant interest (Krueger, 2006).

Reviewer #1 – Data collection

Indicate whether field notes or memos were kept and how they informed the analysis. Thank you for this comment. For your information, data consisted of the transcribed interview, we didn’t use field notes or memos.

Reviewer #1 – Data analysis

Describe how disagreement among coders was resolved. Thank you for this comment. The section has been expanded in relation to the authors' collaboration in the analysis process.

Researcher triangulation was conducted by the first and last author to validate the analysis in phase 4-5. Coded data were used as a basis for reflective discussions between the first and last author about content and meaning, and consensus was reached through these discussions to achieve a nuanced understanding of the data.

The themes were not understood as emerging directly from the data but were actively generated through the researchers’ interpretative engagement with patterns of shared meaning across the coded dataset.

Reviewer #1 – Data analysis

Explain the link between the initial codes and the final 3 themes (the appendix helps but could use a clearer narrative in the results). Thank you for this constructive comment. The link between the initial codes and the final 3 themes has been made explicit in the beginning of the result section:

Three themes were developed through the interpretative process based on 12 codes: 1) Video consultations must be used at the right time, was developed from codes describing how nurses assessed the appropriateness and potential v

Attachment Submitted filename: Response to reviewers.docx

# Peer review: Decision Letter 1

**DOI:** 10.1371/journal.pone.0339752.r003

**Authors:** Nagendra Rai

22 Jul 2026

Exploring nurses’ perspectives on video consultations and the nurse–patient relationship: Findings from qualitative focus group interviews in a Danish cardiology outpatient setting

PONE-D-25-65052R1

Dear Dr. Groth,

We’re pleased to inform you that your manuscript has been judged scientifically suitable for publication and will be formally accepted for publication once it meets all outstanding technical requirements.

Within one week, you’ll receive an e-mail detailing the required amendments. When these have been addressed, you’ll receive a formal acceptance letter and your manuscript will be scheduled for publication.

An invoice will be generated when your article is formally accepted. Please note, if your institution has a publishing partnership with PLOS and your article meets the relevant criteria, all or part of your publication costs will be covered. Please make sure your user information is up-to-date by logging into Editorial Manager at Editorial Manager® and clicking the ‘Update My Information' link at the top of the page. For questions related to billing, please contact billing support.

If your institution or institutions have a press office, please notify them about your upcoming paper to help maximize its impact. If they’ll be preparing press materials, please inform our press team as soon as possible -- no later than 48 hours after receiving the formal acceptance. Your manuscript will remain under strict press embargo until 2 pm Eastern Time on the date of publication. For more information, please contact onepress@plos.org.

Kind regards,

Nagendra Kumar Rai, Ph.D

Guest Editor

PLOS One

Additional Editor Comments (optional):

Dear Authors,

Thank you for your careful and comprehensive revision of the manuscript. I appreciate the considerable effort invested in addressing the comments raised during the previous review.

The revised manuscript demonstrates substantial improvement. The research gap and study aim are now more clearly articulated, the methodological descriptions have been strengthened, the rationale for the hermeneutic approach has been clarified, and the reporting has been improved through greater transparency regarding sampling, data collection, ethical considerations, and analytical procedures. The results now provide greater interpretive depth, and the discussion better contextualizes the findings within the existing literature while appropriately acknowledging the study's limitations. The conclusion has also been strengthened with clearer practical and organizational implications.

Overall, I believe that the authors have satisfactorily addressed the major concerns raised during peer review. The remaining minor issues relate primarily to stylistic or editorial preferences and do not affect the scientific quality of the work.

I have no further substantive comments and recommend the manuscript for publication.

Thanks

Reviewers' comments:

Reviewer's Responses to Questions

**Comments to the Author**

Reviewer #1: All comments have been addressed

**********

2. Is the manuscript technically sound, and do the data support the conclusions?

Reviewer #1: Yes

**********

3. Has the statistical analysis been performed appropriately and rigorously?

Reviewer #1: Yes

**********

4. Have the authors made all data underlying the findings in their manuscript fully available?

The PLOS Data policy

Reviewer #1: Yes

**********

5. Is the manuscript presented in an intelligible fashion and written in standard English?

Reviewer #1: Yes

**********

Reviewer #1: The authors have revised the manuscript as per the comments given to them in the previous review. The content seems fine for acceptance.

**********

what does this mean?). If published, this will include your full peer review and any attached files.

If you choose “no”, your identity will remain anonymous but your review may still be made public.

**Do you want your identity to be public for this peer review?**  For information about this choice, including consent withdrawal, please see our Privacy Policy

Reviewer #1: No

**********

# Editor report: Acceptance letter

**DOI:** 10.1371/journal.pone.0339752.r004

**Authors:** Nagendra Rai

PONE-D-25-65052R1

PLOS One

Dear Dr. Groth,

I'm pleased to inform you that your manuscript has been deemed suitable for publication in PLOS One. Congratulations! Your manuscript is now being handed over to our production team.

At this stage, our production department will prepare your paper for publication. This includes ensuring the following:

* All references, tables, and figures are properly cited

* All relevant supporting information is included in the manuscript submission,

* There are no issues that prevent the paper from being properly typeset

You will receive further instructions from the production team, including instructions on how to review your proof when it is ready. Please keep in mind that we are working through a large volume of accepted articles, so please give us a few days to review your paper and let you know the next and final steps.

Lastly, if your institution or institutions have a press office, please let them know about your upcoming paper now to help maximize its impact. If they'll be preparing press materials, please inform our press team within the next 48 hours. Your manuscript will remain under strict press embargo until 2 pm Eastern Time on the date of publication. For more information, please contact onepress@plos.org.

You will receive an invoice from PLOS for your publication fee after your manuscript has reached the completed accept phase. If you receive an email requesting payment before acceptance or for any other service, this may be a phishing scheme. Learn how to identify phishing emails and protect your accounts at https://explore.plos.org/phishing.

If we can help with anything else, please email us at customercare@plos.org.

Thank you for submitting your work to PLOS ONE and supporting open access.

Kind regards,

PLOS ONE Editorial Office Staff

on behalf of

Dr. Nagendra Kumar Rai

Guest Editor

PLOS One
